# An ultra-dense linkage map identified quantitative trait loci corresponding to fruit quality- and size-related traits in red goji berry

**DOI:** 10.3389/fpls.2024.1390936

**Published:** 2024-09-04

**Authors:** Fazal Rehman, Haiguang Gong, Yun Ma, Shaohua Zeng, Danmin Ke, Chao Yang, Yuling Zhao, Ying Wang

**Affiliations:** ^1^ State Key Laboratory of Plant Diversity and Specialty Crops, Guangdong Provincial Key Laboratory of Applied Botany, Key Laboratory of National Forestry and Grassland Administration on Plant Conservation and Utilization in Southern China, South China Botanical Garden, Chinese Academy of Sciences, South China National Botanical Garden, Guangzhou, China; ^2^ College of Life Science, Gannan Normal University, Ganzhou, Jiangxi, China; ^3^ College of Advanced Agricultural Sciences, University of Chinese Academy of Sciences, Beijing, China; ^4^ Jinghe County Goji Industrial Development Center, Jinghe County, Xinjiang Uygur Autonomous Region, China

**Keywords:** *Lycium*, bitterness and sweetness, soluble solid content, QTL mapping, fruit size/weight, whole genome resequencing, fruit firmness, fruit shape

## Abstract

Goji berries are a small-fruited shrub with industrial importance whose fruit considered beneficial in both fresh and dried forms. Current germplasms of goji berries include small fruits with a short shelf life, less sweet and bitter taste, and a lack of appropriate genetic information. This study aimed to employ whole genome resequencing to generate an ultra-dense bin linkage map and to elucidate the genetic basis of goji fruit quality and size using quantitative trait loci (QTL) mapping analysis in a cross-pollinated hybrid population. To achieve this goal, human sensory tests were carried out to determine the bitter taste (BT) and sweet taste (ST), and to quantify the soluble solid content (SSC), fruit firmness (FF), and fruit size-related traits of fresh goji fruits over three or four years. The results revealed that the goji bin linkage map based on resequencing spanned a total length of 966.42 cM and an average bin interval of 0.03 cM. Subsequent variant calling and ordering resulted in 3,058 bins containing 35,331 polymorphic markers across 12 chromosomes. A total of 99 QTLs, with individual loci in different environments explaining a phenotypic variance of 1.21-16.95% were identified for the studied traits. Ten major effects, including colocalized QTLs corresponding to different traits, were identified on chromosomes 1, 3, 5, 6, 7, and 8, with a maximum Logarithm of Odds (LOD) of 29.25 and 16.95% of explained phenotypic variance (PVE). In addition, four stable loci, one for FF, one for fruit weight (FW), and two for fruit shape index (FSI), were mainly mapped on chromosomes 5, 6, and 7, elucidating 2.10-16.95% PVE. These findings offer valuable insights into the genetic architecture of goji fruit traits along with identified specific loci and markers to further improve and develop sweeter, less bitter and larger fruited goji berry cultivars with extended shelf life.

## Introduction

*Lycium* species are known in ancient China as one of the best botanicals to promote health and longevity. Historically and currently, several species have been used, including *L. barbarum*, followed by *L. chinense*, which predominates the international market (Yao et al., 2018). In China, *Lycium* species, particularly *L. chinense* and *L. ruthenicum*, display morphological, organoleptic, chemical, and pharmacological properties similar to those of *L. barbarum*, also known as goji, and should be considered medicinally comparable. Goji berries are regarded as a new kind of “superfruit” and have progressively made their way into both domestic and international fruit markets because of their distinct taste, nutritional, and health advantages (Kafkaletou et al., 2017; Huang et al., 2021). Different varieties of fresh goji berries differ significantly in physio-chemical and sensory characteristics (Zao et al., 2018). The chemical components of mature goji berry fruit include taurine, alkaloids, polysaccharides, amino acids, trace minerals, vitamins, and volatile compounds (Zhang et al., 2016). The fundamental flavors of sweetness, sourness, and bitterness are produced by these compounds, along with smells that may enhance or detract from the flavor (Fan et al., 2021; Wang et al., 2023). Fresh and semi-fresh goji berries (*L. barbarum*) are moist, chewy, and have a saponaceous flavor, in addition to their original sweet flavor. However, the aftertaste of *L. chinense* berries was slightly sweeter and more bitter than that of *L. barbarum* berries. It has been observed that goji berries from *L. barbarum* are twice as sweet, less sour, bitter, astringent, and saltier than those from *L. chinense*. The greatest difference between the two species was in their sweetness, followed by variations in their sour and bitter flavors (Lee et al., 2014). Goji berries taste like raspberries, blueberries, or blackcurrants and cranberries, but with a hint of tartness and less sweetness. Goji berries have a combination of bitter and sweet flavors, though the strength of each flavor may vary according to the consumer’s palate. To make goji berries easier for consumers to accept, previous studies have shown that they should have characteristics such as a large grain size, bright color, moderate skin thickness, sweeter with less bitter taste, moderate crispness and hardness, fewer seeds and thicker flesh, and more juicy and sweet taste in their fresh variety (Lee et al., 2014). Physical and sensory quality characteristics, including fruit firmness, flavor, and aroma, are critical parameters of goji fruit and are affected during the complex physiological process of fruit ripening (Pech et al., 2018; Fatchurrahman et al., 2022). Fruit quality is a highly complex trait governed by physiological, biochemical, and molecular processes and is quantitatively controlled by polygenes, the growing environment, and their interactions (Sun et al., 2020; Mashilo et al., 2022). The genetic mechanism regulating the fruit quality traits of goji berries has yet to be explored and could facilitate the generation of multiple differentially expressed genes corresponding to goji fruit quality. It has been learned that the fruit size/weight and shape is quantitative trait of goji berries and a major breeding goal, and current varieties of goji berries exhibit small fruit size; for example, *L. barbarum* fruit size is approximately 2.5 cm with a round and oblong shape compared to Chinese goji berries (Rehman et al., 2020). Two well-researched quantitative characteristics of tomatoes are their fruit size and shape. Tanksley et al. (1981) and Paterson et al. (1988) reported that tomato was among the first species for which quantitative trait loci were mapped using a molecular linkage map and discovered more than 30 fruit size and shape loci (Tanksley and Fulton, 2007). Some major-effect fruit size/weights and shape controlling loci have been reported and cloned particularly *fw2.2* (Frary et al., 2000; Guo et al., 2010), *ovate* (Tanksley and Fulton, 2007), *fw3.2* (Chakrabarti et al., 2013), *fw11.3* (Mu et al., 2017), *FASCIATED* (*YABBY2*) (Cong et al., 2008), *WUSCHEL* (*locule number*) and brough significant impact on the fruit size of tomato under genetic and QTL mapping studies (Rodríguez-Leal et al., 2017; Rehman et al., 2022). Fruit shape (length/diameter) loci “*ovate*” discovered near *fw2.2* on chromosome 2 and expressed before anthesis in tomato and allelic variation affects fruit shape index and narrow constriction (Tanksley and Fulton, 2007).

**Figure 1:**
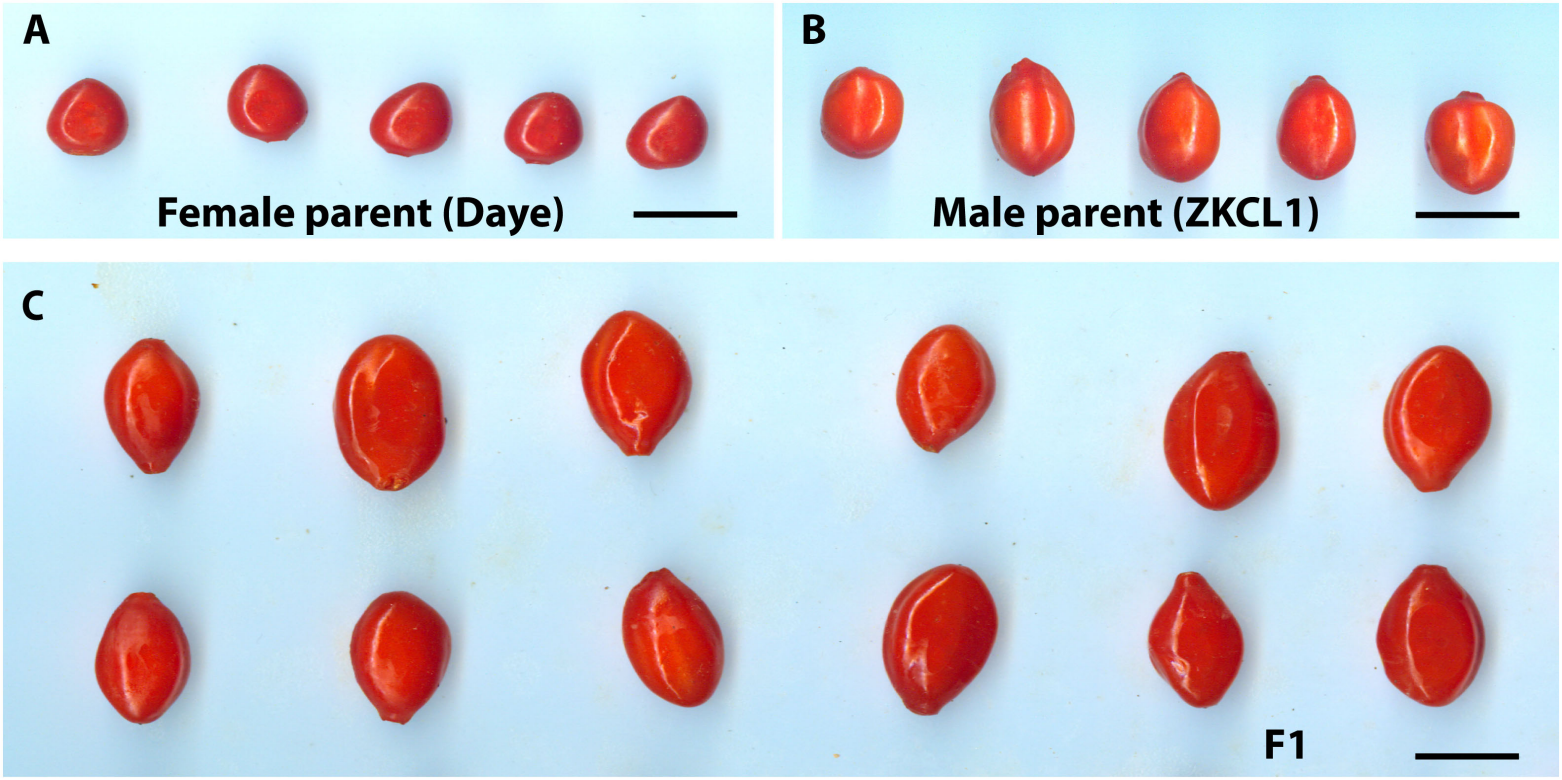
Goji fruits appearance of parents and F_1_ hybrid progeny. **(A)** Fruits of female parent Chinese goji cv. Daye (*Lycium chinense*); **(B)** Fruits of male parent *Lycium barbarum* cv. ZKLC1; **(C)** Randomly chosen F_1_ individual fruits with significant differences in fruit shape and sizes, bars = 1 cm.

Next-generation sequencing (NGS) is a reliable and effective method of genomic mapping and genotyping (Rehman et al., 2020). Using a multiplexed approach, the latest sequencing techniques allow for simultaneous sequencing of numerous samples on a large scale (Craig et al., 2008; Kim et al., 2023). Technological advancements have enabled the development of a sequencing-based high-throughput genotyping technique that combines the benefits of high mapping accuracy and resolution, dense marker coverage, time and cost effectiveness, and comparable genome and genetic maps among mapping populations and organisms (Huang et al., 2009). In recent years, significant advancements have been made in genomic research and sequencing of wolfberry (*L. barbarum*) (Cao et al., 2021). Quantitative trait loci (QTL) of relevant agronomic parameters have been discovered, and multiple high-density genetic maps have been created using single nucleotide polymorphisms (SNPs) and simple sequence repeat (SSR) markers (Gong et al., 2019; Zhao et al., 2019; Rehman et al., 2020; Zhao et al., 2021; Yue et al., 2022). Here, we outlined a high-throughput genotyping technique that uses an ultra-dense bin linkage map of goji berries and SNPs found using whole genome resequencing. QTL mapping was performed to elucidate the genetic basis for physio-chemical and morphological traits, including human sensory tests (sweetness and bitterness), soluble solid content (°brix percentage), fruit firmness, fruit weight, length, diameter, and shape index, from fully matured fruits of the F_1_ individuals of the interspecific population for three or four consecutive years (**Figure 1**). Subsequently, major effect, stable, and overlapping genomic regions, including minor effect loci, were identified with the highest LOD of 29.25 and 16.95% PVE, corresponding to the studied traits. To the best of our knowledge, this is the first study to address goji fruit sweetness, bitterness, °brix percentage, and fruit firmness in relation to the QTL study. This will provide a significant basis for uncovering the genetic architecture of goji fruit quality and size-related parameters, thereby advancing the genetic breeding of goji berries to yield large fruited cultivars with longer shelf-life and less bitter and sweet flavors.

## Materials and methods

### Mapping population and collection of fruit samples

An interspecific F_1_ hybrid population derived from a cross of Daye (*L. chinense*) as the female parent and ZKLC1 (*L. barbarum*) as the male parent (Rehman et al., 2020) was used in the present study. Of the 450 hybrid progenies, 305 were chosen at random in September 2018 and were used to create a genetic linkage map and QTL mapping study of fruit size and quality-related characteristics. The botanical features of the female parent Daye, also referred to as Chinese goji (*L. chinense*), include ovate to lanceolate leaves, shallow to dark purple flowers, an oblong fruit shape, fruit color ranging from dark red to orange, fruit weight (0.38 g), fruit length (1.08 cm), fruit diameter (0.75 cm), and seeds that are generally broader, rounded, and yellowish to yellow-brown in color. The male parent, ZKLC1 (*L. barbarum*), has round fruit with a diameter of 1.28 cm, weight of 1.05 g, length of 1.79 cm, and color of light-yellow seeds that are semi-spherical to flattish. It also has lanceolate to oblong leaves and vivid to royal purple flowers (Rehman et al., 2020). Fully mature fruits of this interspecific hybrid population in the summer season were used as the experimental materials. Fruits of 305 F_1_ individuals and their parents were collected (50 fruits from each tree of similar size and maturity were selected) and stored in a low-temperature refrigerator at 4°C.

### Human sensory tests

Goji fruit tasting evaluation was performed in the laboratory at room temperature at the Northwest China Bio-agricultural Center (38°28′05″ N, 106°16′23″ E), Yinchuan City, Ningxia Hui Autonomous Region, China. Thirty bitter and sweet sensory evaluation forms were prepared, including the name of the participant, gender, age, and a scale for bitterness from 1 (Tasteless) to 7 (extremely bitter) and similarly for sweetness from 1 (Tasteless) to 7 (extremely sweet). In addition, we arranged materials such as mineral water, tasteless cookies, and spittoons for each evaluator. For the taste survey, we invited 30 people (male and female) aged 20 to 60 to evaluate the goji fruit taste from 7:30 to 11:30 AM and from 14:00 to 18:00 PM and completed it on the same day. The taste assessment process was as follows. First, a staff member selected fruit samples from an F_1_ individual and distributed one of its fruits to each individual. The taster then chews the fruit completely in his or her mouth to fill it, spits it out into a spittoon, and rates the sweetness and bitterness within one minute. Finally, gargle with mineral water and get ready to taste the fruit of the next individual and so on. Each person from the panel of 30 people had to rate the goji fruit taste (sweetness and bitterness) for all 305 samples on the same day. Consecutively, red goji fruit taste data were evaluated for three years (2020–2022) during the summer season by the same panel of 30 people each year.

### Determination of physio-chemical traits

Five fruits of the same maturity were chosen for each F_1_ individual and their parents, and the soluble solid content (SSC) (°brix percentage) was measured using a digital pocket refractometer ATAGO PAL-1 (Atago Co. Ltd., Tokyo, Japan). The SSC data were collected consecutively for four years (2019–2022). The results are expressed as °brix percentage. Whereas fruit firmness (FF) was also determined for the five mature fruit repeats per hybrid progeny using the fruit hardness tester equipment GY-4 (Zhejiang Top Instrument Co., Ltd. Hangzhou, China) (Rehman et al., 2020), and data were collected consecutively for three years (2019–2021). The operation of the instrument involves positioning each fruit horizontally on the base and applying gentle, uniform pressure with the pressure head of the penetrometer into the flesh of the fruit. The sensor then displays the reading on the digital panel, and the average value of five consecutive measurements is obtained for each hybrid progeny. Following completion of each set of readings, the scale was reset to zero. Here, we used one-year SSC and FF (2019) data from our previous study (Rehman et al., 2020), and newly collected SSC (2020–2022) and FF (2020–2021) from the F_1_ interspecific hybrid population of red goji berries to re-analyze and update the QTL mapping results based on the reference genome of goji berries (Cao et al., 2021).

### Morphological traits

The morphological data of 305 F_1_ individuals of fruit size-related traits, such as fruit weight (FW) in grams, fruit length (FL), fruit diameter (FD) in centimeters, and fruit shape index (FSI) (determined by dividing fruit length by diameter), were consecutively collected for three years (2018–2020) and statistically analyzed. Data were collected as previously described (Rehman et al., 2020). In the current study, we used two years of data (2018–2019) of four traits (FW, FL, FD, and FSI) from our previous study (Rehman et al., 2020), along with another year (2020) of data to re-analyze and update the QTL mapping results using the goji reference genome (Cao et al., 2021).

### Statistical analysis

Analysis of variance (ANOVA) for three or four individual years, Pearson correlation, and general descriptive statistical analysis, including frequency distribution, were carried out using Origin Pro v. 2024, OriginLab Corporation, Northampton, MA, USA. (https://www.originlab.com/).

### Re-sequencing data analysis and variant calling

The 305 hybrid progenies and parents’ young leaves were collected and dried using a desiccant (silica gel) before DNA extraction. Using a slight modification of the manufacturer’s instructions, DNA from each leaf was extracted using the Plant Genomic DNA Kit (TIANGEN BIOTECH (BEIJING), CHINA, CO., LTD.). The quantity and quality of extracted DNA samples were assessed using a Thermo Fisher Scientific NanoDrop ND-2000 spectrophotometer and 1% agarose gel electrophoresis, respectively (Rehman et al., 2020). Resequencing was performed according to a standard protocol established by Illumina. The genomic DNA of each sample was subjected to library construction once it passed the detection. Those that passed quality control were subjected to the Illumina HiSeq platform for further sequencing. To ensure data quality, raw reads were filtered to obtain paired-end sequencing data following the steps of data filtering, such as the removal of joint sequences contained in reads, using fastp software v. 0.23.0 (Chen et al., 2018); the low-quality bases among reads were removed (the average mass number was calculated using a sliding window of 4bp, and all bases were removed below 15); reads must be greater than 50bp in length. MEM algorithm of the BWA software v. 0.7.15-r1140 (Li, 2013), was first used to compare and align the filtered reads with in-house data along with the *L. barbarum* reference genome with a genome size of 1.68 Gb (Cao et al., 2021) (only the paired-end reads that matched both ends were considered suitable for genome comparison). The SAM tools v. 1.3.1 (Li et al., 2009) was used to exclude duplicate reads from the aligned reads. Genomic variations in each accession were determined using the Genome Analysis Toolkit (GATK) v. 3.7 software’s Haplotype Caller module and GVCF model (McKenna et al., 2010). All GVCF files were merged. Using the Haplotype Caller module, we filtered variants including SNPs and InDels based on strict filtering criteria. The selection criteria were as follows: (a) at least one parent was heterozygous, (b) depth for parents ≥5, (c) depth for individual ≥5, (d) the proportion of missing data in the hybrid progeny did not exceed 25%, and (e) a minor allele frequency (MAF) of less than 10% (Catchen et al., 2011). The SNPs and InDels that were identified were combined and turned into bin markers (Qi et al., 2021). These bin markers were then divided into separate genomic regions and genes, and further annotated using ANNOVAR v. 2016 Feb 1st (Wang et al., 2021) to predict gene function. The region of the mutation site in the genome (intergenic region, gene region, CDS region, etc.) and the impact of the mutation (synonymous and non-synonymous mutations, etc.) were determined based on the location of the mutation site in the reference genome and gene location information on the reference genome.

### Bin linkage map construction and QTL mapping analysis

The construction of the linkage map harboring filtered bin markers was facilitated by the use of Lep-MAP3 software with default parameters, which is based on the maximum likelihood method as described by Rastas (2017). To ensure the precision of the genetic map, bins less than 5kb were initially filtered out, and bins that deviated from the expected Mendelian segregation with a significance level of *P* < 0.001 were excluded from the analysis. The total length of the genetic maps was estimated using the Kosambi mapping function initially proposed by Kosambi (1943). This function is commonly employed to convert recombination frequencies to map distances in centiMorgans (cM). The Order-Marker2 module was used to sort the markers within each linkage group, calculate the genetic distance of each LG, and verify the continuity of the generated maps. A suite of Python MadMapper scripts was used to evaluate the quality of the labeled bin markers on the genetic linkage maps (https://cgpdb.ucdavis.edu/XLinkage/MadMapper/). To assess the quality of the genetic linkage map, a heat map of recombination rates between markers, haplotype map for each hybrid progeny, and collinearity map of the genetic map and genome were generated using CIRCOS v. 0.66 (Krzywinski et al., 2009). GACD software v. 1.2.13 (Zhang et al., 2015), was used for QTL or genomic region identification based on the inclusive composite interval mapping (ICIM) model (Broman and Speed, 2002; Li et al., 2008). The significant threshold was manually set at 2.5 for LOD scores, with 1.0 cM as the walking speed for all QTLs and 0.001 as the PIN value (Wang, 2009; Zhou et al., 2024). QTLs of the eight traits associated with bitter taste (BT), sweet taste (ST), soluble solid content (°brix percentage) (SSC), fruit firmness (FF), fruit weight (FW), fruit length (FL), fruit diameter (FD), and fruit shape index (FSI) were detected across three or four environments over a period of three- or four-years data. A major QTL was considered a QTL with an LOD value > 3 and a phenotypic variance of > 10% across several environments, whereas a stable QTL was found in at least two or three separate environments (Fan et al., 2017; Che et al., 2018). If the confidence intervals overlapped, the QTLs for identical traits found in the other environments were deemed to be the same. The possible locations of the QTLs were described based on the LOD peak sites and surrounding regions. According to the computed results by GACD, which have been thoroughly explained by Nzuki et al. (2017), the additive (a) and dominance (d) effects were calculated using the Muchero formulation (Muchero et al., 2013). The QTL mode of action was determined by dividing dominance by the absolute additive value (d/|a|) ratio. A ratio greater than one was considered over-dominant, a ratio between 0 and 1 as partial dominance, and a ratio less than 1 as under-dominance (Hua et al., 2003).

## Results

### Goji fruit physio-chemical and morphological traits variability analysis

**Figure 2:**
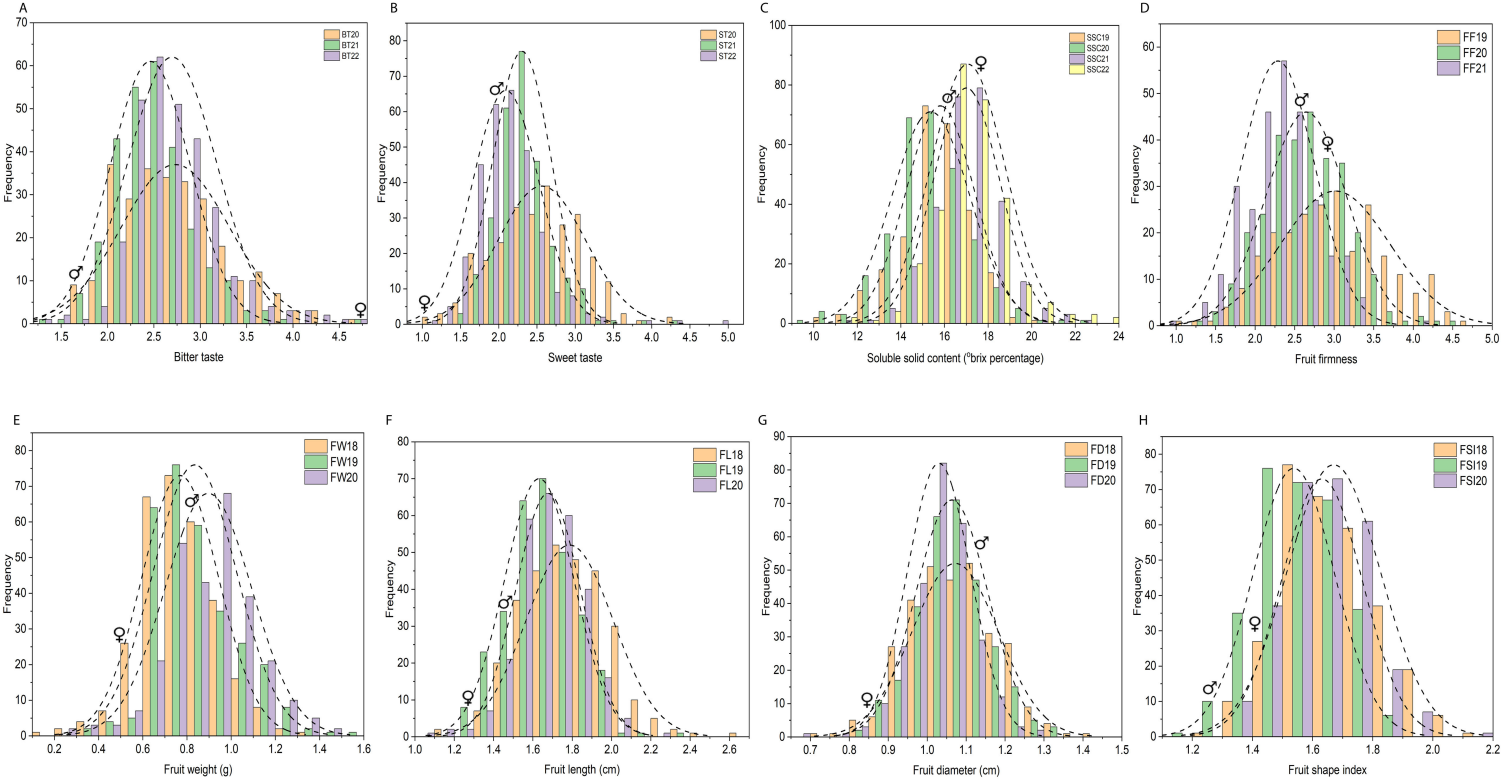
Frequency distribution histogram of 305 F_1_ individuals for physio-chemical and morphological traits based on three or four individual years. **(A–H)** Frequency distribution histogram of 305 F_1_ individuals for bitter taste **(A)**, sweet taste **(B)**, soluble solid content (°brix percentage) **(C)**, fruit firmness (FF) **(D)**, fruit weight (FW) **(E)**, fruit length (FL) **(F)**, fruit diameter (FD) **(G)**, fruit shape index (FSI) **(H)**, based on three or four individual years. Each x-axis represents the value of the trait and the y-axis shows the number of individuals corresponding to the value on the x-axis. BT20, BT21, BT22 bitter taste (2020, 2021, 2022), ST20, ST21, ST22 sweet taste (2020, 2021, 2022), SSC19, SSC20, SSC21, SSC22, soluble solid content (°brix percentage) (2019, 2020, 2021, 2022); FF19, FF20, FF21, fruit firmness (2019, 2020, 2021); FW18, FW19, FW20, fruit weight (2018, 2019, 2020); FL18, FL19, FL20, fruit length (2018, 2019, 2020); FD18, FD19, FD20, fruit diameter (2018, 2019, 2020); FSI18, FSI19, FSI20, fruit shape index (2018, 2019, 2020);♀, indicate female parent location on histogram, ♂, male parent location on histogram.

**Figure 3:**
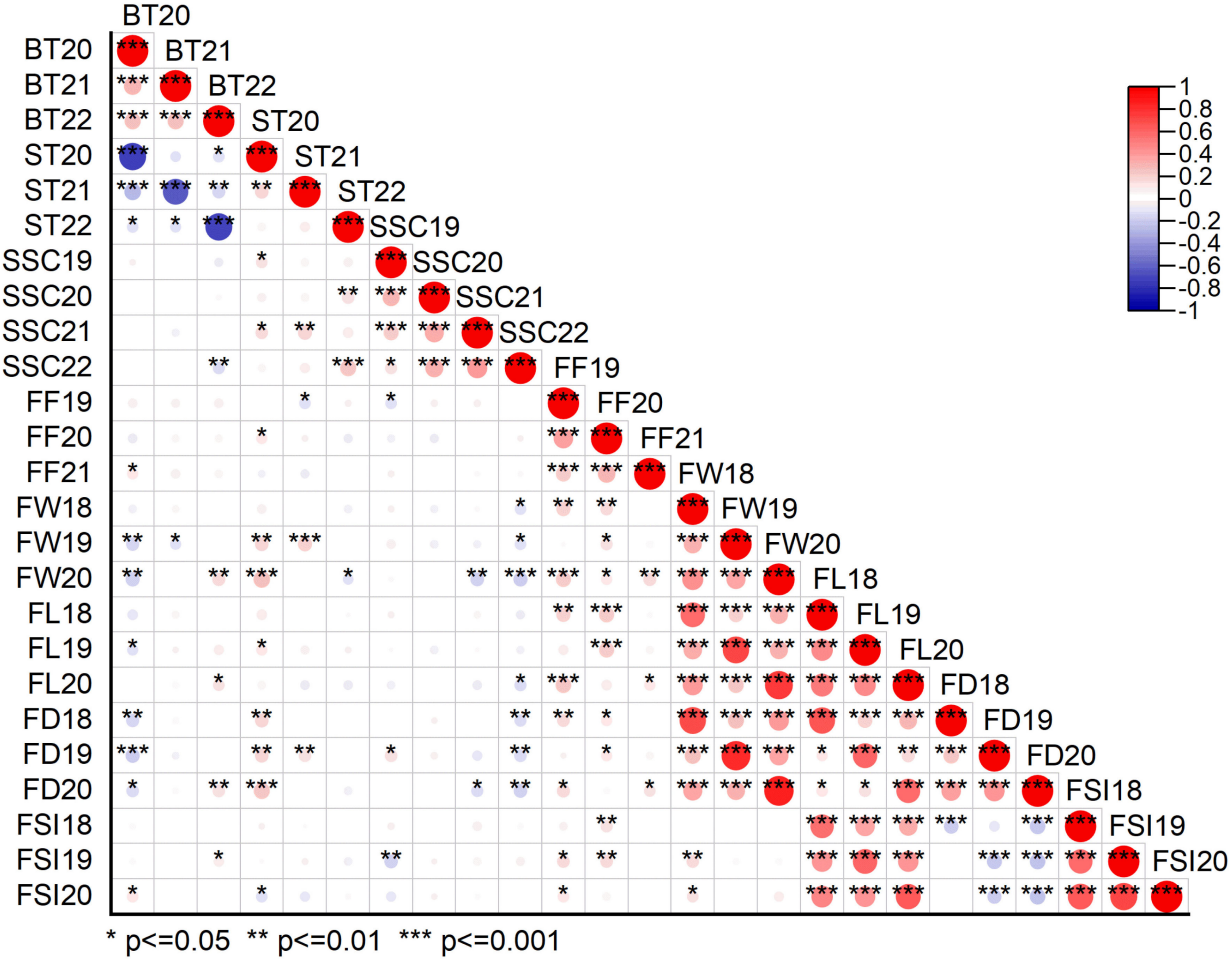
Pearson correlation matrix plot showing the relationship between different physio-chemical and morphological traits under three or four individual years data. BT20, BT21, BT22 bitter taste (2020, 2021, 2022), ST20, ST21, ST22 sweet taste (2020, 2021, 2022), SSC19, SSC20, SSC21, SSC22, soluble solid content (°brix percentage) (2019, 2020, 2021, 2022); FF19, FF20, FF21, fruit firmness (2019, 2020, 2021); FW18, FW19, FW20, fruit weight (2018, 2019, 2020); FL18, FL19, FL20, fruit length (2018, 2019, 2020); FD18, FD19, FD20, fruit diameter (2018, 2019, 2020); FSI18, FSI19, FSI20, fruit shape index (2018, 2019, 2020).

Fruit quality and fruit size/weight related traits, including ST, BT, SSC, FF, FW, FL, FD, and FSI, were evaluated from 305 F_1_ hybrid progeny of interspecific population based on three or four individual years. Analysis of variance (ANOVA) of the studied traits showed significant differences (*P* < 0.05, *P* < 0.01) among F_1_ progenies in different years (**Supplementary Table 1**). Descriptive statistical analysis showed that the coefficient of variation (CV%) ranged from 16 to 22 for ST, 17 to 21 for BT in three years (2020–2022), 8–11 for SSC in four years (2019–2022), 19–22 for FF in three years (2019–2021), 21–22 for FW, 9–12 for FL, 7–10 for FD, 8–9 for FSI in three years (2018–2020) (**Supplementary Table 2**). The frequency distribution histogram and box chart of all eight traits for individual years showed a normal distribution and significant differences among the different years, respectively (**Figures 2A–H**; **Supplementary Figures 1A–H**). Normality tests were performed using the Kolmogorov-Smirnov (K-S) goodness of fit based on the absolute distance between the cumulative distribution and values ranged from 0.02 to 1, suggesting a positive normal distribution of all traits evaluated under different years except BT21, BT22, ST22, SSC22, and FW19 (**Supplementary Table 3**). Correlation analysis (*P* < 0.05 or *P* < 0.01) revealed an extremely significant positive association detected in a few comparisons among BT and FW, FD; ST and SSC, FW, FD; SSC and FD; FF and FW, FL, FD, FSI; and fruit size related traits such as fruit weight, fruit length, fruit diameter and fruit shape index showed highly positive significant correlation among each other. A highly significant negative correlation was also detected between BT and ST, FW, FD; SSC and FSI, FW, FL, FD; and FD and FSI (**Supplementary Table 4**; **Figure 3**).

### Whole genome resequencing (WGS) and genotyping

**Figure 4:**
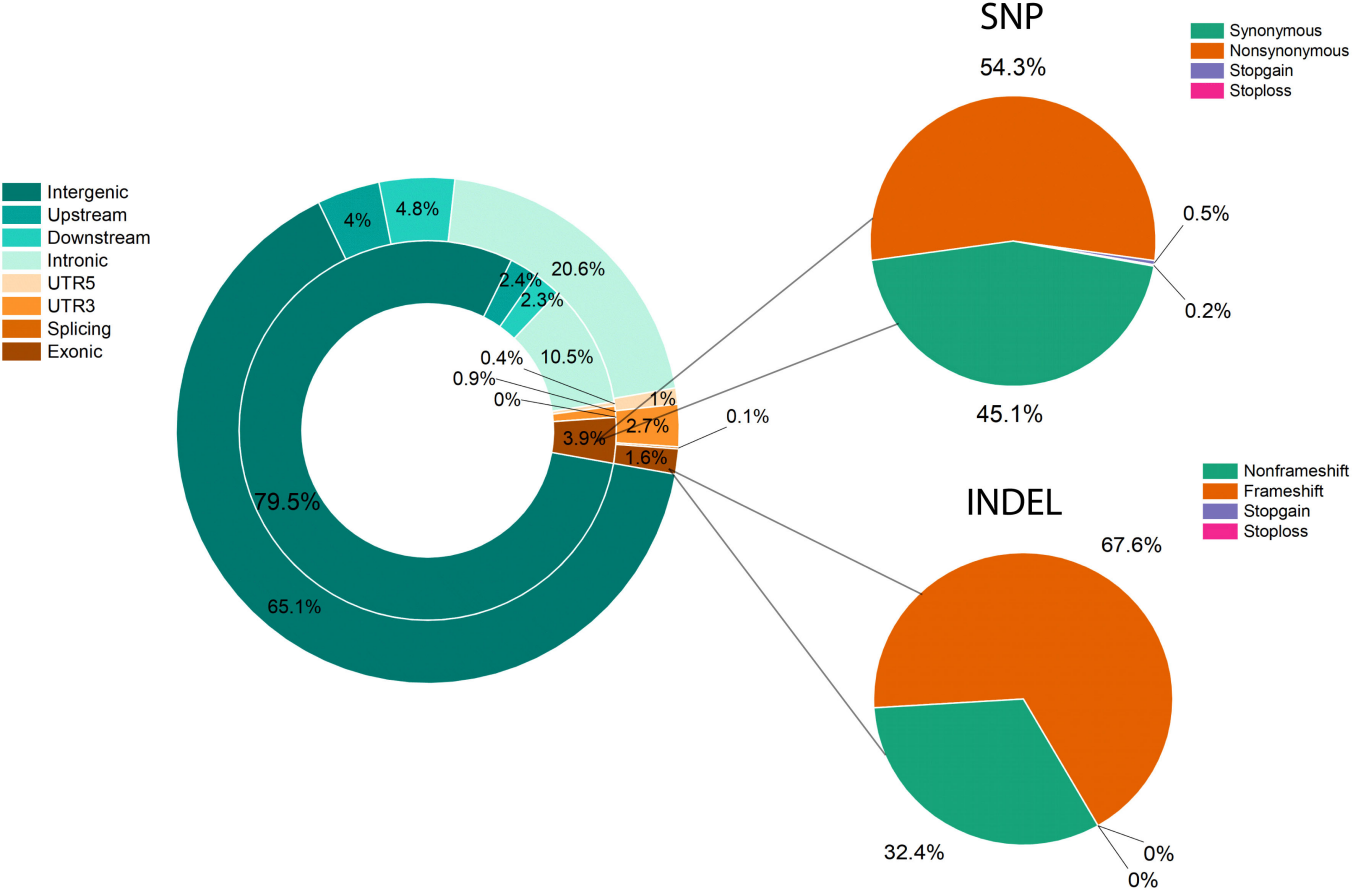
Variant distribution and annotation for SNPs and InDels. Genome annotation and functional annotation of SNPs and InDels. The left doughnut diagram is the result of the genome annotation, the inner circle is the SNP, and the outer circle is InDel. The right pie chart is the functional annotation for SNP (top) and InDels (bottom).

Resequencing was performed for raw read data of the two parents and 305 F_1_ individuals using *in-house* data and the *L. barbarum* reference genome (Cao et al., 2021). As shown in **Supplementary Table 5**, approximately 36.27 and 29.67 million cleaned reads from parents (DY and ZKLC1) and an average of 9.46 million cleaned reads from F_1_ individuals including 2.9 billion total clean reads, were obtained. The average GC content was 39.85% with Q20 and Q30 scores ranged from 98.91 to 96.54%. The average sequencing coverage and depth of DY, ZKLC1, and F_1_ individuals were 5.31%, 49.33; 8.09%, 43.93; and 4.61%, 17.57; respectively. Of these reads, 50.62% from the female parent (DY), 82.19% from the male parent (ZKLC1), and 63.96% from F_1_ individual plants were uniquely mapped onto the reference genome of *L. barbarum* and employed for SNP calling (**Supplementary Table 5**). Finally, 3,058 bins containing total 35,331 polymorphic markers between the two parents were obtained, comprising 33,212 total SNPs with SNP density of 239.01 SNP/Mb, and 2,119 InDels with InDel density of 15.38 InDel/Mb. SNPs and InDels were distributed across the genome, as illustrated in Supplementary (**Supplementary Figure 2**). Detailed information on these SNPs is provided in Supplementary (**Supplementary Table 6**). Most annotated SNPs (79.5%) were detected in intergenic areas, and 3.9% were located in the exonic region. Of these, 54.3% were nonsynonymous SNPs. Compared to SNPs, intergenic regions accounted for over half (65.1%) of the annotations for InDels, with exonic regions accounting for 1.6% of the total. Frameshift mutations resulted in 67.6% exon InDels (**Figure 4**).

### Goji integrated bin map construction and evaluation

Goji bin linkage map comprising 12 chromosomes were constructed harboring 3,058 bins containing 35,331 polymorphic markers, that varied from 197 to 377 bins and spanned a coverage ranged from 56.22 to 109.33 cM. The integrated genetic map spans a total length of 966.42 cM comprising 847.73 cM and 1065.89 cM lengths of female parent and male parent, respectively. The mean interval between bins were 0.03 cM with an average bin interval of 0.319 cM, and an average of maximum distance between the bins were 4.223 cM (**Supplementary Figure 3**). The longest chromosome was Ch06 (Lba06) spanning a length of 109.33 cM and comprising 2,947 markers in 377 bins. Ch02 (Lba02), on the other hand, was the smallest chromosome, spanning 56.22 cM and having 197 bins with 5,823 markers (**Supplementary Table 7**). The 1,121 bins on the female genetic map had an average maximum bin interval of 7.363 cM and an average spacing between the markers and bins of 0.027 and 0.786 cM, respectively. In contrast, the male genetic map had 1,966 bins, with an average maximum bin interval of 2.616 cM and an average spacing between the markers and bins of 0.033 and 0.551 cM, respectively (**Supplementary Table 7**). The quality of the constructed consensus genetic map was evaluated using haplotype maps (**Supplementary Figure 4**) and heatmaps (**Supplementary Figure 5**) of 305 F_1_ individuals based on 12 linkage groups. The results indicated that the bin genetic map was constructed with high quality and good collinearity corresponding to goji genome. To assess collinearity between the genetic map and the genome, 3,058 bins were mapped to the reference genome of the goji berries. For all 12 LGs, correlations between the genetic and physical maps were nearly linear (**Supplementary Figure 6**). The order of the majority of bins in the linkage map matched that of the corresponding chromosomes in the physical map of the goji berry genome. **Supplementary Table 8** presents the correlation coefficients for each LG. For all LGs, the average correlation coefficient between physical and genetic positions was 0.86. These LGs exhibited a high degree of genetic collinearity with the physical map, as demonstrated in our findings. Significant collinearity indicated that the markers adequately covered the genome of goji berries and appropriately covered the 12 chromosomes.

### Identification of QTLs using the ultra-dense linkage map

**Figure 5:**
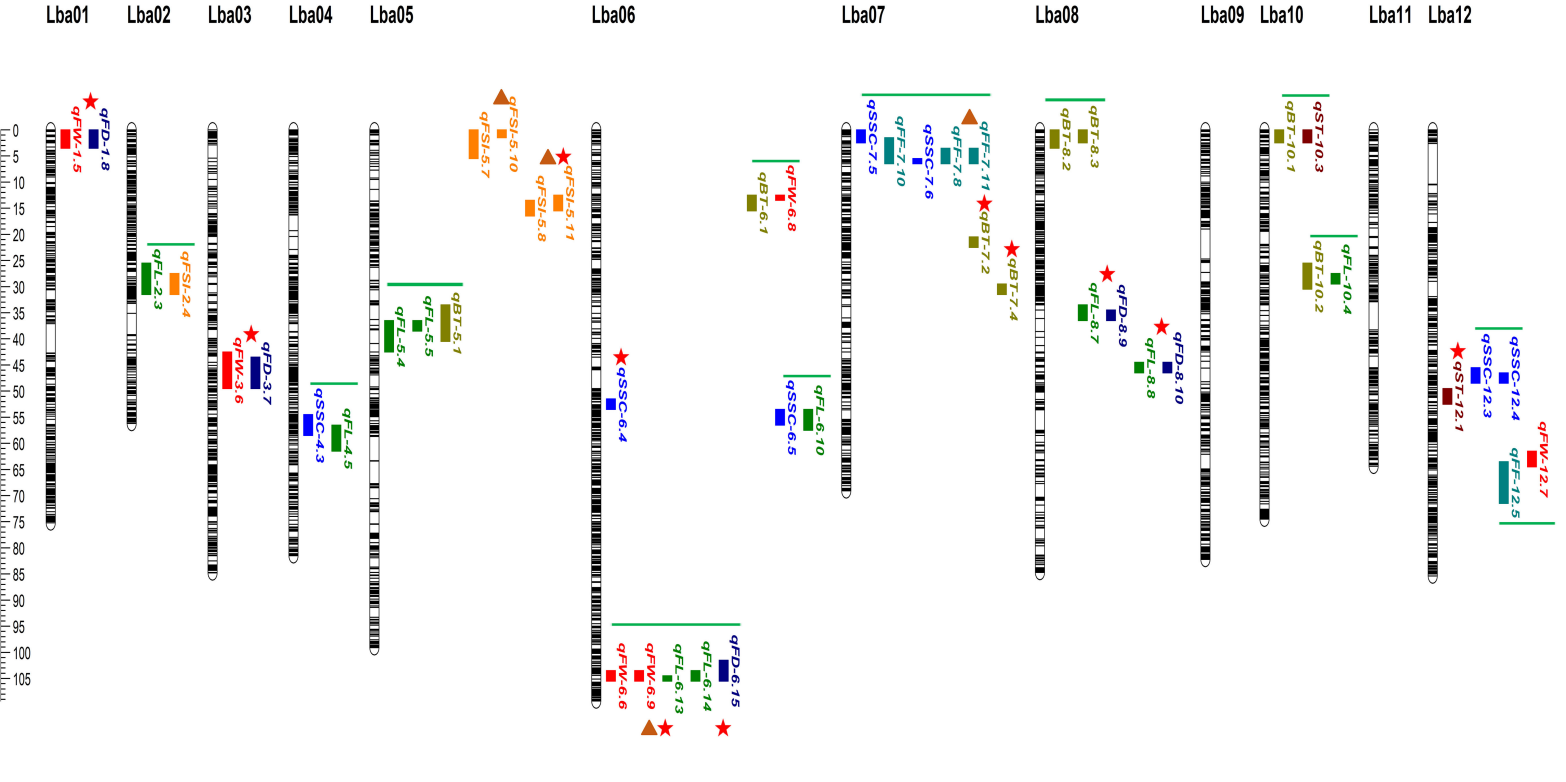
The ultra-dense bin linkage map of goji berry and distribution of major-effect, stable and overlapped QTLs corresponding to physio-chemical and morphological traits identified under three or four individual years. Different vertical color tiles represent various traits QTLs. Maroon, *qST*_sweet taste; olive, *qBT*_bitter taste; blue, *qSSC*_soluble solid content (°brix percentage); sea green, *qFF*_fruit firmness; red, *qFW*_fruit weight; green, *qFL*_fruit length; dark blue, *qFD*_fruit diameter; orange, *qFSI*_fruit shape index. Red star indicates major-effect and colocalized QTLs, brown triangle represents stable QTLs, and green horizontal bars represent overlapped QTLs.

Using the ultra-dense genetic map of goji berries, a number of QTLs were plotted for physio-chemical and morphological traits based on three or four consecutive years of data. QTL mapping analysis was performed for BT, ST SSC, FF, FW, FL, FD, and FSI using the ICIM model in GACD v. 1.2.13. Most QTLs associated with the studied traits were located on distinct chromosomes, including chromosomes 3, 4, 5, 6, 7, 8, 9, and 12 (**Figure 5**; **Supplementary Table 9**). A total of Ninety-nine QTLs targeting eight traits were found on separate chromosomes in the individual years (**Supplementary Table 9**). Ten major effect and colocalized QTLs were detected on chromosomes 1, 3, 5, 6, 7, and 8, whereas stable QTLs targeting different traits were detected across two different environments (**Figure 5**; **Table 1**). QTLs detected corresponding to each trait across three or four environments are described as follows:

**Table 1:** All major-effect, and stable QTLs corresponding to the studied traits identified in three or four individual years.

Traits	QTLs	Year	Chromosome	Position (cM)	Flanking markers	LOD	PVE (%)	*a*	*d*	*d*/|*a*|	CI (cM)
BT	*qBT-7.2*	2021	7	21	Marker13033977-Marker13326504	29.25	16.95	-0.895	-0.045	0.051	20.5–22.5
	*qBT-7.4*	2021	7	30	Marker19870129-Marker19934823	26.87	16.13	0.910	-0.013	-0.015	29.5–31.5
ST	*qST-12.1*	2020	12	50	Marker83874194-Marker84016091	14.12	10.34	0.731	-0.022	-0.030	49.5–52.5
SSC	*qSSC-6.4*	2021	6	52	Marker95684790-Marker95762010	15.24	11.50	0.992	0.121	0.122	51.5–53.5
FW	*qFW-1.5*	2018	1	2	Marker3398563-Marker4171036	11.54	10.39	0.814	-0.061	-0.075	0–3.5
FD	*qFD-1.8*	2018	1	2	Marker3398563-Marker4171036	3.88	2.46	0.03	-0.02	-0.60	0–3.5
FW	*qFW-3.6*	2019	3	45	Marker127088931-Marker127359380	4.55	5.91	-0.068	-0.032	0.476	42.5–49.5
FD	*qFD-3.7*	2019	3	45	Marker127088931-Marker127359380	4.46	5.36	-0.03	-0.01	0.46	43.5–49.5
FW	*qFW-6.6*	2018	6	105	Marker127994636-Marker127996736	3.13	2.62	-0.035	-0.063	1.808	103.5–105.5
FW	*qFW-6.9*	2020	6	105	Marker127994636-Marker127996736	2.74	3.64	-0.152	0.501	-3.293	103.5–105.5
FL	*qFL-6.13*	2020	6	105	Marker127994636-Marker127996736	4.48	5.97	-0.54	0.61	-1.13	104.5–105.5
FL	*qFL-8.7*	2018	8	36	Marker30212799-Marker30212822	5.77	5.44	-0.206	-0.016	0.078	33.5–36.5
FD	*qFD-8.9*	2018	8	36	Marker30212799-Marker30212822	6.93	5.72	-0.10	0.00	0.043	34.5–36.5
FL	*qFL-8.8*	2018	8	45	Marker33160675-Marker33394294	9.55	10.80	0.641	0.030	0.047	44.5–46.5
FD	*qFD-8.10*	2018	8	45	Marker33160675-Marker33394294	17.08	12.25	0.87	0.02	0.023	44.5–45.5
FSI	*qFSI-5.8*	2018	5	15	Marker6172058-Marker6899663	9.71	6.38	-0.721	-0.040	0.056	13.5–16.5
	*qFSI-5.11*	2020	5	15	Marker6172058-Marker6899663	9.28	7.64	-0.082	-0.007	0.081	13.5–15.5
FF	*qFF-7.8*	2019	7	5	Marker4744364-Marker5267296	2.69	2.11	-0.016	-0.046	2.796	3.5–6.5
	*qFF-7.11*	2021	7	5	Marker4744364-Marker5267296	3.21	1.27	-0.037	-0.100	2.726	3.5–6.5
FSI	*qFSI-5.7*	2018	5	1	Marker2562548-Marker3019144	3.33	2.10	0.032	-0.010	-0.322	0–5.5
	*qFSI-5.10*	2020	5	1	Marker2562548-Marker3019144	5.08	3.97	0.041	-0.015	-0.370	0–1.5

BT, bitter taste; ST, sweet taste; SSC, soluble solid content; FF, fruit firmness; FW, fruit weight; FL, fruit length; FD, fruit diameter; FSI, fruit shape index; Position, The scanning position in cM on the chromosome; Flanking markers, left marker of the current scanning position and right marker of the current scanning position; LOD, LOD score; PVE (%), Phenotypic variation explained by QTL at the current scanning position; *a*, additive effects of parents; *d*, dominance effects of parents; *d*/|*a*|, QTL mode of action; CI (cM), Left and Right Confidence interval calculated by one-LOD drop from the estimated QTL position (cM).

**Bitter Taste (BT):** Seventeen QTLs targeting BT were detected on different chromosomes (**Supplementary Table 9**), including two major QTLs (*qBT-7.1, qBT-7.2*) explaining the total phenotypic variance of 16.95%, 16.13%, and LOD scores of 29.25, 26.87, and positioned on chromosome 7 on the map with 20.5–21.5, 29.5–31.5, and flanking the marker interval (Marker13033977-Marker13326504; Marker19870129-Marker19934823) (**Figure 5**; **Table 1**). Five loci (*qBT-5.1, qBT-8.2, qBT-10.1, qBT-10.2*, and *qBT-6.1*) targeting BT overlapped with (*qFL-5.4-qFL-5.5, qBT-8.3, qST-10.3, qFL-10.4*, and *qFW-6.8*), harboring a maximum PVE of 5.22% with 7.75 LOD and coincided confidence intervals (**Figure 5**; **Supplementary Table 9**). The major QTLs showed positive and negative additive effects, indicating a large contribution from parents with partial dominance effects of the QTLs (**Table 1**).

**Sweet Taste (ST):** Twelve ST QTLs were detected on different chromosomes (**Supplementary Table 9**), including one major-effect (*qST-12.1*) and five minor-effect (*qST-6.2, qST-10.3, qST-11.1, qST-11.2*, and *qST-12.2*) QTLs were detected with the highest LOD score of 14.12 and PVE of 10.34% (**Figure 5**; **Table 1**; **Supplementary Table 9**). The additive effect of major loci showed a positive contribution from both parents, with the partial-dominance effect of the QTL (**Table 1**).

**Soluble Solid Content (SSC) (°brix percentage):** Sixteen genomic regions corresponding to SSC were located on different chromosomes (**Supplementary Table 9**), including *qSSC-6.4*, a major-effect locus, with an LOD score of 15.24 and 11.50% variance. Additionally, 11 QTLs (*qSSC-4.3, qFL-4.5*; *qSSC-6.5*, *qFL-6.10*; *qSSC-12.3, qSSC-12.4*; *qSSC-7.5, qFF-7.10*; *qSSC-7.6, qFF-7.8, qFF-7.11*) were detected as overlapping loci corresponding to different traits with a maximum LOD of 7.25 and 6.22% phenotypic variance (**Figure 5**; **Supplementary Table 9**).

**Fruit Firmness (FF):** A total of 10 FF QTLs were detected on different chromosomes with LOD ranged from 2.52–7.53 and PVE was 1.27–7.08% across the three environments (**Supplementary Table 9**). A stable locus (*qFF-7.8-qFF-7.11*) was identified with an LOD score of 5.90 and phenotypic variance of 3.38 across two environments and showed negative additive effect of parents with an over-dominance effect of QTL (**Figure 5**, **Table 1**). *qFF-12.5* was found to overlap with *qFW-12.7* harboring maximum LOD of 7.79 and phenotypic variance 5% (**Supplementary Table 9**).

**Fruit size related traits:** Thirteen FW QTLs were identified (**Supplementary Table 9**), including three major-effect QTLs (*qFW-1.5, qFD-1.8*; *qFW-3.6, qFD-3.7*; *qFW-6.6, qFW-6.9, qFL-6.13*) accounting for LOD scores of 11.42, 9.01, and 10.35, and PVE of 12.85%, 11.27%, and 12.23%, respectively, and flanking the marker intervals (M3398563-M4171036; Marker127088931-Marker127359380; M127994636-M127996736), respectively (**Figure 5**). These three major loci exhibited positive and negative effects on parents, indicating partial and over-dominance effects of QTLs, respectively (**Table 1**). A stable locus (*qFW-6.6, qFW-6.9, qFL-6.13*) also overlapped with one colocalized locus (*qFL-6.14, qFD-6.15*) across the two environments, along with similar flanking marker and confidence intervals (**Figure 5**; **Table 1**). The additive effect of the stable QTL was negative for parents, indicating over-dominance effects of the QTL (**Table 1**). For FL, 16 loci were detected on different chromosomes (**Supplementary Table 9**), including two major-effect and co-localized QTLs (*qFL-8.7, qFD-8.9*; *qFL-8.8, qFD-8.10*), accounting for LOD scores of 12.70, 26.63, and PVE of 11.16% and 23.05% and flanking the marker intervals (Marker30212799-Marker30212822; Marker33160675-Marker33394294), exhibited negative and positive additive effects with partial dominance effects of QTL (**Figure 5**, **Table 1**). *qFL-2.3*, and *qFL-5.4*, were observed to overlap with *qFSI-2.4*, and *qFL-5.5*, with a maximum LOD score of 4.59 and a variance of 2.34 (**Figure 5**; **Supplementary Table 9**). Eight FD QTLs were detected on various chromosomes (**Supplementary Table 9**), including *qFD-8.8*, a major-effect locus with an LOD of 27.08, and phenotypic variance of 14.25% (**Figure 5**, **Table 1**). Seven FSI QTLs were detected on different chromosomes (**Supplementary Table 9**), including one major QTL (*qFSI-5.7-qFSI-5.10*) and two stable QTLs (*qFSI-5.6-qFSI-5.9*; *qFSI-5.7-qFSI-5.10*) with LOD values of 8.41 and 18.99, and phenotypic variance of 6.07% and 14.06%, respectively, flanking the marker interval (Marker2562548-Marker3019144; Marker6172058-Marker6899663) (**Figure 5**, **Table 1**). The additive effects of major and stable QTLs were negative and positive for parents, respectively, and showed partial and under-dominance effects of QTLs (**Table 1**). The stable and overlapping genomic regions are particularly important loci because of their extreme positive correlation and pleiotropic effects, and could be considered for the identification of potential candidate genes corresponding to fruit bitterness, sweetness, firmness, and fruit size/weight of goji berries.

## Discussion

### Ultra-dense linkage map of goji berry

In this study, the whole genome resequencing (WGS) approach was employed on the two parents and interspecific 305 F_1_ individuals and re-analyzed using *in-house* databases along with the *L. barbarum* reference genome. WGS of plant genomes offers profound insights into genome evolution and enables the discovery of a vast array of DNA markers, including SNPs, InDels, copy number variations, and presence/absence variations in crops. By improving the accuracy, output, speed, and affordability of genome-wide genotyping, WGS-based methods can transform QTL mapping (Huang et al., 2009; Xu and Bai, 2015; Pan et al., 2022);. Building an ultra-dense linkage map with precise genotyping using polymorphic markers such as SNPs has become feasible with the advancement of NGS technologies and the release of the goji berry reference genome. Nevertheless, there is a report of a goji berry high-density genetic map containing 15,240 SNP markers based on resequencing, and identified leaf- and fruit-related trait loci (Zhao et al., 2021). Similar studies utilizing a large number of SNP markers in different crops have reported the use of WGS to generate high-density genetic maps (Li et al., 2018; Guo et al., 2023). This will offer useful resources for marker-assisted selection, map-based gene cloning, and candidate-gene discovery. Our research presents an incredibly detailed genetic map of the goji berry, which was derived from whole-genome resequencing of both F_1_ individuals and parental lines. The WGS method applied in this study for constructing genetic maps is notable for its integration of genotyping, SNP validation, and discovery (Xu and Bai, 2015). In our previous study, SLAF-seq was used to identify 3,495 SNP markers that were subsequently used to build a genetic map of goji without a reference genome (Rehman et al., 2020). To identify recombination breakpoints, we sequenced the parental lines at 49-fold and 43-fold sequencing depths, and the average sequencing depth of the F_1_ individuals was 17-fold, yielding high-quality clean reads. Comparatively, the average depths in the female (NN) and male (YG) parents and F_1_ individuals were 34-fold, 18-fold, and 2.58-fold, respectively (Zhao et al., 2021). Unmatched recombination intervals from previous genotypic data (Rehman et al., 2020) were filtered, and an ultra-dense genetic map was built using bins derived from reliable genotypic data. Following genotyping analysis, 12 linkage groups were assigned to 3,058 recombination bins, corresponding to 35,331 polymorphic markers. A previous study identified 8,507 SNP markers to create a consensus genetic map for goji berries (Zhao et al., 2021). Our findings demonstrate the effectiveness of the WGS strategy for both ultra-dense linkage map building and marker discovery, which accurately reflect the features of genomic and genetic variation in goji berries. Bin genetic mapping offers several benefits, including the ability to overcome the constraints imposed by conventional mapping techniques and capture the entire recombination landscape of a mapping population by selecting the most relevant markers from a large pool of marker datasets (Guan et al., 2022). During map construction, consistent markers paired as recombinant bins decreased marker placement faults and genetic distance estimation errors, while avoiding duplicate loci. This technique has proven effective in producing ultra-dense genetic linkage maps across a range of species (Li et al., 2011; Chen et al., 2014; Ma et al., 2016; Qin et al., 2022). Recently, several studies have constructed genetic maps of goji berries using NGS-based methods (Gong et al., 2019; Zhao et al., 2019; Rehman et al., 2020; Zhao et al., 2021; Yue et al., 2022). Gong et al. (2019) published a high-density genetic linkage map of goji berries using the double-digest restriction site-associated DNA (ddRAD) of genotyping by sequencing (GBS) method. The map contained 2,764 bins spanning a total length of 964.03 cM, with an average distance of 0.038 cM between adjacent bins. A high-density genetic map with a total genetic distance of 1702.45 cM was created using large-scale SLAF-seq for SNPs detection (Zhao et al., 2019). Using whole genome resequencing, Zhao et al. (2021) constructed an SNP-based consensus genetic linkage map with 12, linkage groups, 15,240 SNP markers, and a total distance spanning 3,058.19 cM with an average marker distance of 0.21. Earlier, a high-density genetic map of goji berry was developed using SLAF-seq, which covered a total distance of 1,649.03 cM and comprised of 3,495 SNP markers with an average interval of 0.47 cM between adjacent markers (Rehman et al., 2020). In the present study, a similar mapping population (Rehman et al., 2020) was employed, and a greater frequency of polymorphisms for map construction and notable variances in a number of features were caused by the large genetic diversity between the two parental lines. Using WGS, 3,058 bin markers including 35,331 polymorphic markers were identified and accommodated into 12 chromosomes The linkage map was 966.42 cm in total length, with an average distance of 0.03 cm between adjacent bins. Our genetic map was populated by a large number of additional SNP markers. In contrast, recent studies have shown that genetic maps of goji berries include fewer SNP markers (Gong et al., 2019; Zhao et al., 2019; Rehman et al., 2020; Zhao et al., 2021). Conversely, the resequencing-based linkage map revealed a smaller comprehensive map distance and a greater total number of SNPs, implying that WGS exhibits higher resolution than reduced-representation sequencing (Rehman et al., 2020). To date, an extremely dense and highly saturated linkage map of *Lycium* has been developed successfully. The collinearity of the genetic and physical maps was uniform for each linkage group, which is consistent with previous reports (Rehman et al., 2020; Zhao et al., 2021). Furthermore, visual assessment of the haplotype and heat maps of the genetic map, along with synteny analysis of the genetic map and reference genome, indicated that our map was produced appropriately and that the F_1_ individuals were eligible for genetic study. This ultra-dense linkage map serves as a strong source for map-based cloning, marker-assisted selection breeding, and fine-mapping of potential genes for significant agronomic and economic traits.

### Discovery of QTLs corresponding to physio-chemical and morphological traits

In light of the significance of the important fruit quality and size/weight attributes of goji berries, phenotypic data of the studied traits were collected consecutively for three or four years. When evaluating data from different years corresponding to the eight traits, correlation analysis revealed a highly negative association between human-evaluated BT and ST. Conversely, a highly positive association was observed between ST and SSC, but no association was detected between ST22 and ST20/ST21, possibly due to the rejection of the null hypothesis for ST22 under normality tests (**Supplementary Table 3**). In addition, fruit firmness was positively associated with fruit weight and length, whereas fruit size-related traits showed a highly positive correlation between different years, except for FD and FSI, with highly negative associations. The extremely negative correlation corresponding to fruit bitterness under different years with fruit sweetness, and SSC with FSI and FW, and highly positive correlation of fruit size-related traits might indicate a tight association among linked markers or even candidate genes due to pleiotropic effects. This information can help to determine candidate gene predictions. The outcomes of the correlation analysis between bitterness, sweetness, and SSC revealed a noticeable disparity between human taste evaluations and the readings obtained from digital instruments, such as refractometers. We posited that human sensory tests for fresh goji berries would exhibit lower precision and accuracy in determining bitterness and sweetness, potentially as a result of variations in taste buds and environmental factors, such as differences in planting areas. However, it is crucial to consider the sugar content determined by a refractometer when evaluating the taste of fresh goji berries. A similar study reported no significant association between color variations and visual judgment scores for bitterness and sweetness (Huang et al., 2021). In sensory evaluation, a negative correlation was found between the color score and bitterness, and a highly positive association was found between the color score and taste evaluation, indicating the influence of color differences on taste evaluation (Huang et al., 2021). The presence of flavonoid compounds with a dihydroquercetin structure is thought to contribute to the bitterness of goji fruit (Yuan et al., 2017). Moreover, the bitterness of wild peach fruits is associated with a higher concentration of phenolic compounds than that in landraces and improved cultivars (Predieri et al., 2005). The sugar-acid ratio and assessment of bitterness were linked to the titratable acid content in goji berry fruit (Huang et al., 2021). An intriguing taste pattern ranged from bitter to half-bitter, bitter after sweet, hemp bitter after sweet, sweet, slightly bitter after sweet, and whole bitter of freshly matured fruits was found in another study that assessed the germplasm of red goji berries (Yuan et al., 2022). The F_1_ populations of Ningqi-7 and wild bitter goji berries displayed proportions of sweetness and bitterness, whereas the reverse cross produced only bitter F_1_ individuals, indicating the heterozygosity of bitterness-related genes, which are controlled by dominant genes with additive genetic effects, whereas recessive genes may be responsible for controlling sweetness (Yuan et al., 2017). The distinct sweetness of *L. barbarum* goji fruit compared to *L. chinense* fruit is attributed to the presence of betaine, a naturally occurring amino acid. Research has demonstrated that higher concentrations of betaine are associated with increased sweetness (Lee et al., 2014).

In the present study, 99 genomic loci corresponding to eight physio-chemical and morphological traits were identified. The highest phenotypic variance explained by all detected QTLs varied from 1.15–16.95% with 2.52–29.25 LOD. The additive and dominance effects of each identified QTL were then quantified. The cumulative effect of an individual allele at a locus influences the trait value, which is known as the additive effect of a QTL. This is the result of the alleles assigned by each parent. The trait value is enhanced by alleles from the mother parent when there is a positive additive effect and enhanced by alleles from the father when there is a negative additive effect (Hu et al., 2021). In contrast, the dominance effect describes how one allele can supersede or mask the effects of another allele at the same locus; moreover, it also defines the interaction between alleles at a specific locus, which leads to deviation from an additive genetic model. Among breeding populations, the additive effect of a QTL is more significant as it directly contributes to the overall genetic variance than dominance effects; thus, it is considered a decisive factor in determining the genetic architecture of complex traits (Zhang et al., 2008). QTL mapping analysis found 17 total BT QTLs, two major loci with negative and positive additive effects and five overlapping genomic regions with the highest LOD scores of 29.25, and phenotypic variance of 16.95%. Similar studies have identified a bitterness QTL (*qbt-c1–1*) with a high LOD score in watermelons (Li et al., 2018) and 27 QTLs for fruit taste in peaches (Li et al., 2019). Two QTLs associated with bitterness (*qBT.1*, and *qBT.2*) were found in bottle gourds LG02 and LG09, respectively (Wu et al., 2019). Similar to earlier reports, goji fruit bitterness-associated loci were identified on chromosomes 1, 2, and 9 (*qBT-1.1, qBT-2.1, qBT-9.1*, and *qBT-9.2*) with a positive additive effect and the highest LOD score of 4.14 (**Supplementary Table 1**). Twelve QTLs targeting ST, including a major-effect locus (*qST-12.1*) and five minor-effect QTLs, had the highest LOD of 14.12 (**Table 1**; **Supplementary Table 9**). Similarly, 21 loci targeting SSC (°brix percentage), including *qSSC-8.6* with the highest phenotypic variance (11.50%), and a positive additive effect were identified as the major locus (**Supplementary Table 9**). Six other SSC loci overlapped with QTLs corresponding to FL and FF. An earlier study identified two fruit sweetness (SSC) QTLs (*qFS3–1* and *qFS5–2*) with relative LODs of 5.48 and 9.2% PVE on LG03 and LG05, respectively (Rehman et al., 2020). However, *qSSC-4.4* was identified (using the same 2019-year data) on chromosome 4, with a PVE of 4.14% and an LOD score of 2.86, exploiting an ultra-dense bin linkage map. Multiple SSC QTLs have been reported (Wu et al., 2021; Miller et al., 2022), including fruit flesh sugar content regulation QTLs (*Qfru2–3, QBRX2–1*, and *QBrix6*) on chromosomes 2 and 6 in watermelons (Sandlin et al., 2012; Ren et al., 2014). Consistent with previous findings, SSC QTLs (*qSSC-2.3, qSSC-6.3, qSSC-6.4*, and *qSSC-6.5*) were also detected on chromosomes 2, and 6 with a maximum LOD score of 27.24 (**Supplementary Table 9**). Fresh fruit firmness is an important quality trait in goji berries that affects fruit texture, and is ultimately associated with shelf life and economic value. Fruit firmness is a quantitative trait regulated by multiple genes (Dai et al., 2022). Ten loci were associated with fruit firmness including a stable locus (*qFF-7.8, qFF-7.13*) (**Figure 5**), and an overlapped loci with *qFF-7.10* on chromosome 7, with LOD score of 3.01, and 5.90, respectively. Two fruit firmness QTLs (*qff2.1*, and *qff5.1*) were detected on chromosomes 2 and 5 in melons with high LOD scores of 3.8, 17.44, 28%, and 38% of variance, respectively (Dai et al., 2022; Chen et al., 2023). Similar investigations have found one locus (*qff4.1*) on chromosome 4 for sweet cherry fruit firmness (Cai et al., 2019) and blueberry fruit firmness QTLs on chromosome 8 (Cappai et al., 2020). One locus (*qFF-5.2*) corresponding to goji fruit firmness was identified on chromosome 5, with an LOD score of 3.02. Previously, a fruit firmness (FF) QTL (*qFF10–1*) was positioned on LG10 with a relative LOD of 4.81 and 8.1% phenotypic variance (Rehman et al., 2020). However, four FF loci (*qFF-4.4, qFF-7.8, qFF-7.9*, and *qFF-12.5*) were identified (using the same 2019-year data), and positioned on chromosomes 4, 7, and 12 with variance ranging from 2.04–7.08% and LOD scores of 2.53–7.53, respectively. It has been learned that the colocalization of QTLs governing various fruit size/weight-related traits provides an unusual opportunity to gain profound insights into the intricate mechanisms underlying fruit size traits. This colocalization results from the pleiotropic effects of particular genes, linkage disequilibrium, or convergence of several important genes within the same genomic area (Kato et al., 2000; Baytar et al., 2021). Moreover, because correlated traits are likely governed by comparable genetic causes, mapping them to similar genomic sites is expected to establish an interesting genetic architecture (Kato et al., 2000). In the present study, major-effect and colocalized QTLs targeting fruit size/weight accounted for a maximum LOD score of 11.54, and phenotypic variance of 12.23% was found to have positive and negative additive effects. Moreover, stable loci (*qFW-6.6, qFW-6.9*, and *qFL-6.13*) displaying negative additive effects were identified across the two environments with similar confidence intervals (**Table 1**). Consistent with our findings, previous studies have reported major fruit weight QTLs (*qAFW6.1, qAFW12.1*) with positive and negative additive effects (Lin et al., 2010; Aruna et al., 2023). Nine fruit weight (FW) QTLs were positioned on LG10 with LOD ranging from 4.72–7.8 and 6.9–11.1% PVE in two individual years (2018–19) (Rehman et al., 2020). However, in the current study, 11 FW loci were identified based on the previous two years (2018–19) data and were positioned on different chromosomes with PVE ranged from 2.36–10.39% and LOD score ranging from 2.58–11.54 (**Supplementary Table 9**). Similarly other goji studies have reported seven fruit weight loci in different linkage groups with a variance of less than 10%, including one stable FW loci on LG10 with a PVE of 59% (Zhao et al., 2019, 2021). Several loci corresponding to fruit length, along with two major- and co-localized loci (*qFL-8.7, qFD-8.9*; *qFL-8.8, qFD-8.10*) showing positive and negative additive effects with a PVE > 10%, were identified on different chromosomes (**Table 1**; **Supplementary Table 9**). Previously, five fruit length (FL) QTLs were positioned on LG10 with LOD ranging from 6.08–8.1 and 8.8–11.5% variance, in two individual years (2018–19) (Rehman et al., 2020); nevertheless, 12 FL loci were identified and positioned on different chromosomes with phenotypic variance ranging from 1.15–11.16% and LOD score ranging from 2.53–22.70 (**Supplementary Table 9**). A similar study on goji found six FL loci on LG10 and LG11 including two stable FL loci (*qFL10* and *qFL12*), which explained up to 36% of the phenotypic variance (Zhao et al., 2019, 2021). Another investigation discovered two major fruit length QTLs on chromosomes 1 and 7 with a PVE of > 10% (Aruna et al., 2023). For fruit diameter Eight QTLs were detected, including a major and colocalized locus (*qFD-8.10, qFL-8.8*) with a phenotypic variance of 12.25%, revealing a positive additive effect (**Table 1**; **Supplementary Table 9**). Earlier, QTL mapping results into five fruit diameter (FD) QTLs were positioned on LG10 with LOD ranging from 4.5–7.8 and 6.6–11.1% PVE in two individual years (2018–19) (Rehman et al., 2020); however, seven FD loci were identified and positioned on different chromosomes with variance ranging from 2.94–12.25% and LOD scores ranging from 2.59–17.08 (**Supplementary Table 9**). Similar findings have identified four fruit width QTLs in different linkage groups, with PVE exceeding 10% for two QTLs (Zhao et al., 2019). QTLs targeting the fruit shape index were identified, with one major QTL (*qFSI-5.8, qFSI-5.11*) and two stable QTLs (*qFSI-5.7, qFSI-5.10*; *qFSI-5.8, qFSI-5.11*) harboring LOD values of 8.41, 18.99, and PVE of 6.07% and 14.02%, respectively. Previously, eight fruit shape index (FSI) QTLs were positioned on different linkage groups with LOD ranged from 4.55–7.08 and 6.6–10.2% PVE in two individual years (2018–19) (Rehman et al., 2020). Comparatively, four FSI loci were identified, which were positioned on chromosomes 2 and 5, with PVE ranging from 2.10–6.38% and LOD score ranged from 3.27–9.71 (**Supplementary Table 9**). A similar study found 24 loci targeting the fruit index (FI) on different linkage groups with a PVE greater than 10%, and two stable QTLs (*qFI7–1* and *qFI7–2*) corresponding to FI were detected in LG07, with a PVE of up to 19% (Zhao et al., 2019, 2021). The colocalization of fruit size QTLs, such as *qFW-1.5, qFD-1.8, qFW-3.6, qFD-3.7*; *qFW-6.6, qFW-6.9, qFL-6.13*; *qFL-8.7, qFD-8.9*; *qFL-8.8, qFD-8.10*; and *qFL-6.14, qFD-6.15*), may be associated with strong positive correlations among fruit weight, length, and diameter. The loci causing FW, FL, and FD were located at the same positions on chromosomes 1, 3, 6, and 8, supporting the high association between these three fruit size traits, suggesting that multiple genetic factors govern each of the three. The intriguing relationship between multiple loci that govern fruit bitterness, sweetness, firmness, SSC, and size/weight QTLs (*qBT-8.2, qBT-8.3, qBT-10.1, qST-10.3, qBT-10.2, qFL-10.4, qBT-6.1, qFW-6.8*; *qFL-5.5, qBT-5.1*; *qSSC-4.3, qFL-4.5*; *qSSC-6.5, qFL-6.10*; *qSSC-12.3, qSSC-12.4*; *qSSC-7.5, 7.6, qFF-7.8, 7.10, 7.11*; *qFF-12.5, qFW-12.7*; and *qFL-2.3, qFSI-2.4*) located on the same chromosomes might indicate a positive correlation, suggesting a functional interrelationship between these genetic regions (**Figure 5**; **Supplementary Table 9**). This could provide a valuable resource for identifying candidate genes related to fruit quality and size in future studies. A similar phenomenon of colocalization and overlapping loci associated with different traits has been reported previously and is consistent with our findings (Xu et al., 2017; Aruna et al., 2023).

## Conclusion

In conclusion, an ultra-dense bin linkage map of goji berries was constructed with 3,058 bins containing 35,331 polymorphic markers, based on the re-analysis of 305 F_1_ individuals of the interspecific population and goji reference genome using WGS. Our ultra-dense bin linkage map was of 966.4 cM a total genetic distance of 0.03 cM with a bin interval and harbored more SNP markers than in recently reported studies in goji berries. Simultaneously, this study introduces human sensory tests of fresh goji berries to assess the bitterness, sweetness, and measurement of °brix percentage, fruit firmness, and fruit size-related traits to decipher the genetic architecture underlying goji taste, firmness mechanism, size/weight, and improve goji breeding in terms of economically important fruit traits. Altogether, 99 QTLs, including ten major-effect and colocalized and four stable QTLs corresponding to the eight physio-chemical and morphological traits, were identified on different chromosomes. Additionally, the SNP markers found within these QTLs can be used to support marker-assisted selection, making it possible to develop more effective and accurate breeding plans for goji berry size/weight, and fruit quality. We anticipate that this study will represent a significant advancement in our understanding of the genetic architecture underlying goji fruit quality- and size-related traits. The comprehensive identification of QTLs, their potential for fine mapping, and the availability of molecular markers for marker-assisted breeding collectively contribute to the broader goal of developing sweeter, less bitter, large-fruited cultivars with extended shelf-life and empowering the goji industry with higher economic gains.

## Data Availability

The datasets presented in this study can be found in online repositories (Wang et al., 2017; Zhang et al., 2020). The names of the repository/repositories and accession number(s) can be found below: https://bigd.big.ac.cn/gsa, CRA002920.

## Supplementary material

Supplementary Figure 1 Box chart of 305 F_1_ individuals for physio-chemical and morphological traits based on three or four individual years. **(A–H)** Box chart diagram of 305 F_1_ individuals BT, ST, SSC, FF, FW, FL, FD, and FSI based on three or four individual years. Each x-axis represents the trait in different years, and the y-axis shows the frequency of ranges corresponding to the value on the x-axis. In each box chart, the lower and upper lines represent the first and third quartiles, respectively, and the middle line represents the median. BT20, BT21, BT22 bitter taste (2020, 2021, 2022), ST20, ST21, ST22 sweet taste (2020, 2021, 2022), SSC19, SSC20, SSC21, SSC22, soluble solid content (°brix percentage) (2019, 2020, 2021, 2022); FF19, FF20, FF21, fruit firmness (2019, 2020, 2021); FW18, FW19, FW20, fruit weight (2018, 2019, 2020); FL18, FL19, FL20, fruit length (2018, 2019, 2020); FD18, FD19, FD20, fruit diameter (2018, 2019, 2020); FSI18, FSI19, FSI20, fruit shape index (2018, 2019, 2020).

Supplementary Figure 2 The distribution of variation across the genome. **(A)** Number and types of single nucleotide polymorphism (SNPs), **(B)** Number and length of Insertion/deletion (InDel).

Supplementary Figure 3 An integrated bin linkage map shows the distribution of polymorphism markers on 12 linkage groups (LGs) of red goji berry. The black bars on each linkage group show markers distribution.

Supplementary Figure 4 Haplotype mapping of F_1_ individuals based on 12 linkage groups in the integrated maps. In each individual map, the horizontal line represents a marker and the column shows a chromosome in a sample. The first column of the map shows a paternal chromosome and the second column shows a maternal chromosome, and individuals are separated by blank columns. Green indicates the first allele from the parent, red indicates the second allele from the parent, white indicates an unjudged event, and gray indicates a missing event. The positions where color changes in the same column display reorganization events.

Supplementary Figure 5 Heatmaps of F_1_ individuals based on 12 linkage groups of the integrated bin linkage map. Each row and column represent a marker arranged in the order of the map. Each small square shows the recombination rate between the two markers. The color change from blue to yellow to red indicates the reorganization rate from small to large. The blue color indicates a closer marker recombination rate, whereas the closer the color from blue to red, farther becomes the recombination rate.

Supplementary Figure 6 Collinearity between the genetic and physical positions on each chromosome.

## References

[B1] Aruna T. S. Srivastava A. Tomar B. S. Behera T. K. Krishna H. Jain P. K.. (2023). Genetic analysis of heat tolerance in hot pepper: insights from comprehensive phenotyping and QTL mapping. Front. Plant Sci. 14. doi: 10.3389/fpls.2023.1232800 PMC10491018 37692444

[B2] Baytar A. A. Peynircioğlu C. Sezener V. Frary A. Doğanlar S. (2021). Molecular mapping of QTLs for fiber quality traits in Gossypium hirsutum multi-parent recombinant inbred lines. Euphytica. 217, 181. doi: 10.1007/s10681-021-02914-9

[B3] Broman K. W. Speed T. P. (2002). A model selection approach for the identification of quantitative trait loci in experimental crosses. J. R. Stat. Soc Ser. B R. Stat. Soc 64, 641–656. doi: 10.1111/1467-9868.00354 PMC2651044 19104078

[B4] Cai L. Quero-García J. Barreneche T. Dirlewanger E. Saski C. Iezzoni A. (2019). A fruit firmness QTL identified on linkage group 4 in sweet cherry (*Prunus avium* L.) is associated with domesticated and bred germplasm. Sci. Rep. 9. doi: 10.1038/s41598-019-41484-8 PMC6428808 30899090

[B5] Cao Y. L. Li Y. L. Fan Y. F. Li Z. Yoshida K. Wang J. Y.. (2021). Wolfberry genomes and the evolution of *Lycium* (Solanaceae). Commun. Bio. 4, 671. doi: 10.1038/s42003-021-02152-8 34083720 PMC8175696

[B6] Cappai F. Amadeu R. R. Benevenuto J. Cullen R. Garcia A. Grossman A.. (2020). High-resolution linkage map and QTL analyses of fruit firmness in autotetraploid blueberry. Front. Plant Sci. 11. doi: 10.3389/fpls.2020.562171 PMC7701094 33304360

[B7] Catchen J. M. Amores A. Hohenlohe P. Cresko W. Postlethwait J. H. (2011). Stacks: building and genotyping loci *de novo* from short-read sequences. G3-Genes Genomes Genet. 1, 171–182. doi: 10.1534/g3.111.000240 PMC3276136 22384329

[B8] Chakrabarti M. Zhang N. Sauvage C. Muños S. Blanca J. Cañizares J.. (2013). A cytochrome P450 regulates a domestication trait in cultivated tomato. Proceed. Nat. Acad. Sci. 110 (42), 17125–17130. doi: 10.1073/pnas.1307313110 PMC3801035 24082112

[B9] Che Y. Song N. Yang Y. Yang X. Duan Q. Zhang Y.. (2018). QTL mapping of six spike and stem traits in hybrid population of Agropyron Gaertn. in multiple environments. Front. Plant Sci. 9. doi: 10.3389/fpls.2018.01422 PMC6218563 30425721

[B10] Chen K. Dai D. Wang L. Yang L. Li D. Wang C.. (2023). SLAF marker-based QTL mapping of fruit-related traits revealed a major-effect candidate locus ff2.1 for flesh firmness in melon. J. Integ. Agric. 22, 3331–3345. doi: 10.1016/j.jia.2023.02.014

[B11] Chen Z. Wang B. Dong X. Liu H. Ren L. Chen J.. (2014). An ultra high-density bin-map for rapid QTL mapping for tassel and ear architecture in a large F 2 maize population. BMC Genomics 15, 1–10. doi: 10.1186/1471-2164-15-433 24898122 PMC4059873

[B12] Chen S. Zhou Y. Chen Y. Gu J. (2018). fastp: an ultra-fast all-in-one FASTQ preprocessor. Bioinformatics 34, i884–i890. doi: 10.1093/bioinformatics/bty560 30423086 PMC6129281

[B13] Cong B. Barrero L. S. Tanksley S. D. (2008). Regulatory change in YABBY-like transcription factor led to evolution of extreme fruit size during tomato domestication. Nat. Genet. 40, 800–804. doi: 10.1038/ng.144 18469814

[B14] Craig D. W. Pearson J. V. Szelinger S. Sekar A. Redman M. Corneveaux J. J.. (2008). Identification of genetic variants using bar-coded multiplexed sequencing. Nat. Methods 5, 887–893. doi: 10.1038/nmeth.1251 18794863 PMC3171277

[B15] Dai D. Zeng S. Wang L. Li J. Peng J. Liu H.. (2022). Identification of fruit firmness QTL ff2.1 by SLAF-BSA and QTL mapping in melon. Euphytica. 218. doi: 10.1007/s10681-022-02999-w

[B16] Fan Z. Hasing T. Johnson T. S. Garner D. M. Schwieterman M. L. Barbey C. R.. (2021). Strawberry sweetness and consumer preference are enhanced by specific volatile compounds. Hortic. Res. 8. doi: 10.1038/s41438-021-00502-5 PMC8012349 33790262

[B17] Fan C. Zhai H. Wang H. Yue Y. Zhang M. Li J.. (2017). Identification of QTLs controlling grain protein concentration using a high-density SNP and SSR linkage map in barley (*Hordeum vulgare* L.). BMC Plant Biol. 17, 122. doi: 10.1186/s12870-017-1067-6 28697758 PMC5504602

[B18] Fatchurrahman D. Amodio M. L. De Chiara M. L. V. Mastrandrea L. Colelli G. (2022). Characterization and postharvest behavior of goji berry (Lycium barbarum l.) during ripening. Post. Biol. Tech. 191, 111975. doi: 10.1016/j.postharvbio.2022.111975

[B19] Frary A. Nesbitt T. C. Frary A. Grandillo S. Knaap E. V. D. Cong B.. (2000). fw2. 2: a quantitative trait locus key to the evolution of tomato fruit size. Science 289 (5476), 85–88. doi: 10.1126/science.289.5476.85 10884229

[B20] Gong H. Rehman F. Yang T. Li Z. Zeng S. Pan L.. (2019). Construction of the first high-density genetic map and QTL mapping for photosynthetic traits in *Lycium barbarum* L. Mol. Breed. 39, 1–13. doi: 10.1007/s11032-019-1000-9

[B21] Guan W. Ke C. Tang W. Jiang J. Xia J. Xie X.. (2022). Construction of a high-density recombination bin-based genetic map facilitates high-resolution mapping of a major QTL underlying anthocyanin pigmentation in eggplant. Inter. J. Mol. Sci. 23, 10258. doi: 10.3390/ijms231810258 PMC9499331 36142175

[B22] Guo T. Qiu Q. Yan F. Wang Z. Bao J. Yang Z.. (2023). Construction of a high-density genetic linkage map based on bin markers and mapping of QTLs associated with fruit size in jujube (*Ziziphus jujuba* mill.). Hort. 9, 836. doi: 10.3390/horticulturae9070836

[B23] Guo M. Rupe M. A. Dieter J. A. Zou J. Spielbauer D. Duncan K. E.. (2010). Cell Number Regulator1 affects plant and organ size in maize: implications for crop yield enhancement and heterosis. Plant Cell. 22 (4), 1057–1073. doi: 10.1105/tpc.109.073676 20400678 PMC2879740

[B24] Hu G. Wang B. Gong T. Li R. Guo X. Liu W.. (2021). Mapping additive and epistatic QTLs for forage quality and yield in soybean [Glycine max (L.) Merri.] in two environments. Biotechnol. Biotechnol. Equip. 35, 839–852. doi: 10.1080/13102818.2021.1932593

[B25] Hua J. Xing Y. Wu W. Xu C. Sun X. Yu S.. (2003). Single-locus heterotic effects and dominance by dominance interactions can adequately explain the genetic basis of heterosis in an elite rice hybrid. Proc. Natl. Acad. Sci. 100, 2574–2579. doi: 10.1073/pnas.0437907100 12604771 PMC151382

[B26] Huang X. Feng Q. Qian Q. Zhao Q. Wang L. Wang A.. (2009). High-throughput genotyping by whole-genome resequencing. Genome Res. 19, 1068–1076. doi: 10.1101/gr.089516.108 19420380 PMC2694477

[B27] Huang T. Yan Y. Liu J. Zhang B. He X. He X.. (2021). Physical and chemical qualities and sensory evaluation of fresh fruits of *Lycium barbarum* L. Food Res. Dev. 42, 19. doi: 10.12161/j.issn.1005-6521.2021.19.004

[B28] Kafkaletou M. Christopoulos M. V. Tsantili E. (2017). Short-term treatments with high CO_2_ and low O_2_ concentrations on quality of fresh goji berries (*Lycium barbarum* L.) during cold storage. J. Sci. Food Agric. 97, 5194–5201. doi: 10.1002/jsfa.8401 28447344

[B29] Kato K. Miura H. Sawada S. (2000). Mapping QTLs controlling grain yield and its components on chromosome 5A of wheat. Theor. Appl. Genet. 101, 1114–1121. doi: 10.1007/s001220051587

[B30] Kim J. Kim S. Yeom H. Song S. W. Shin K. Bae S.. (2023). Barcoded multiple displacement amplification for high coverage sequencing in spatial genomics. Nat. Commun. 14 5261. doi: 10.1038/s41467-023-41019-w 37644058 PMC10465490

[B31] Kosambi D. D. (1943). The estimation of map distances from recombination values. Ann. Hum. Genet. 12, 172–175. doi: 10.1111/j.1469-1809.1943.tb02321.x

[B32] Krzywinski M. Schein J. Birol I. Connors J. Gascoyne R. Horsman D.. (2009). Circos: an information aesthetic for comparative genomics. Genome Res. 19, 1639–1645. doi: 10.1101/gr.092759.109 19541911 PMC2752132

[B33] Lee H. W. Kim Y. H. Kim Y. H. Lee G. H. Lee M. Y. (2014). Discrimination of *Lycium chinense* and *Lycium barbarum* by taste pattern and betaine analysis. Inter. J. Clin. Exper. Med. 7, 2053–2059. PMC4161546 25232386

[B34] Li H. (2013). Aligning sequence reads, clone sequences and assembly contigs with BWA-MEM. arXiv preprint arXiv:1303.3997. doi: 10.6084/M9.FIGSHARE.963153.V1

[B35] Li Y. Cao K. E. Zhu G. Fang W. Chen C. Wang X.. (2019). Genomic analyses of an extensive collection of wild and cultivated accessions provide new insights into peach breeding history. Genome Bio. 20, 1–18. doi: 10.1186/s13059-019-1648-9 30791928 PMC6383288

[B36] Li H. Handsaker B. Wysoker A. Fennell T. Ruan J. Homer N.. (2009). The sequence alignment/map format and SAMtools. Bioinformatics 25, 2078–2079. doi: 10.1093/bioinformatics/btp352 19505943 PMC2723002

[B37] Li B. Lu X. Dou J. Aslam A. Gao L. Zhao S.. (2018). Construction of a high-density genetic map and mapping of fruit traits in watermelon (Citrullus lanatus L.) based on whole-genome resequencing. Inter. J. Mol. Sci. 19, 3268. doi: 10.3390/ijms19103268 PMC6214002 30347873

[B38] Li H. Ribaut J. Li Z. Wang J. (2008). Inclusive composite interval mapping (ICIM) for digenic epistasis of quantitative traits in biparental populations. Theor. Appl. Genet. 116, 243–260. doi: 10.1007/s00122-007-0663-5 17985112

[B39] Li W. Zhang J. Mou Y. Geng J. McVetty P. B. Hu S.. (2011). Integration of Solexa sequences on an ultradense genetic map in *Brassica rapa* L. BMC Genomics 12, 1–14. doi: 10.1186/1471-2164-12-249 PMC3125265 21595929

[B40] Lin K. H. Yeh W. L. Chen H. M. Lo H. F. (2010). Quantitative trait loci influencing fruit-related characteristics of tomato grown in high-temperature conditions. Euphytica 174, 119–135. doi: 10.1007/s10681-010-0147-6

[B41] Ma X. Fu Y. Zhao X. Jiang L. Zhu Z. Gu P.. (2016). Genomic structure analysis of a set of *Oryza nivara* introgression lines and identification of yield-associated QTLs using whole-genome resequencing. Sci. Rep. 6, 27425. doi: 10.1038/srep27425 27251022 PMC4890301

[B42] Mashilo J. Shimelis H. Ngwepe R. M. Thungo Z. (2022). Genetic analysis of fruit quality traits in sweet watermelon (Citrullus lanatus var. lanatus): A review. Front. Plant Sci. 13, 834696. doi: 10.3389/fpls.2022.834696 35392511 PMC8981301

[B43] McKenna A. Hanna M. Banks E. Sivachenko A. Cibulskis K. Kernytsky A.. (2010). The Genome Analysis Toolkit: a MapReduce framework for analyzing next-generation DNA sequencing data. Genome Res. 20, 1297–1303. doi: 10.1101/gr.107524.110 20644199 PMC2928508

[B44] Miller B. A. Kostick S. A. Luby J. J. (2022). Large-effect QTLs for titratable acidity and soluble solids content validated in ‘Honeycrisp’-derived apple germplasm. Agr. 12, 1703. doi: 10.3390/agronomy12071703

[B45] Mu Q. Huang Z. Chakrabarti M. Illa-Berenguer E. Liu X. Wang Y.. (2017). Fruit weight is controlled by cell size regulator encoding a novel protein that is expressed in maturing tomato fruits. PloS Genet. 13 (8), e1006930. doi: 10.1371/journal.pgen.1006930 28817560 PMC5560543

[B46] Muchero W. Sewell M. M. Ranjan P. Gunter L. E. Tschaplinski T. J. Yin T.. (2013). Genome anchored QTLs for biomass productivity in hybrid Populus grown under contrasting environments. PloS One 8, e54468. doi: 10.1371/journal.pone.0054468 23382900 PMC3558514

[B47] Nzuki I. Katari M. S. Bredeson J. V. Masumba E. Kapinga F. Salum K.. (2017). QTL mapping for pest and disease resistance in cassava and coincidence of some QTL with introgression regions derived from Manihot glaziovii. Front. Plant Sci. 8. doi: 10.3389/fpls.2017.01168 PMC5519584 28785268

[B48] Pan L. Wang M. Yang Y. Chen C. Dai H. Zhang Z.. (2022). Whole-genome resequencing identified QTLs, candidate genes and Kompetitive Allele-Specific PCR markers associated with the large fruit of Atlantic Giant (*Cucurbita maxima*). Front. Plant Sci. 13. doi: 10.3389/fpls.2022.942004 PMC9354748 35937359

[B49] Paterson A. H. Lander E. S. Hewitt J. D. Peterson S. Lincoln S. E. Tanksley S. D. (1988). Resolution of quantitative traits into Mendelian factors by using a complete linkage map of restriction fragment length polymorphisms. Nat. 335, 721–726. doi: 10.1038/335721a0 2902517

[B50] Pech J. Purgatto E. Bouzayen M. Latché A. (2018). Ethylene and fruit ripening. Ann. Plant Rev. Online, 275–304. doi: 10.1002/9781119312994.apr0483

[B51] Predieri S. Ragazzini P. Rondelli R. (2005). Sensory evaluation and peach fruit quality. In VI International Peach Symposium. Acta Hortic. 713, 429–434. doi: 10.17660/ActaHortic.2006.713.63

[B52] Qi X. P. Ogden L. E. Bostan H. Sargent J. D. Ward J. Gilbert J.. (2021). High-density linkage map construction and QTL identification in a diploid blueberry mapping population. Front. Plant Sci. 12. doi: 10.3389/fpls.2021.692628 PMC8256855 34234801

[B53] Qin M. F. Li L. T. Singh J. Sun M. Y. Bai B. Li S. W.. (2022). Construction of a high-density bin-map and identification of fruit quality-related quantitative trait loci and functional genes in pear. Hortic. Res. 9, uhac141. doi: 10.1093/hr/uhac141 36072841 PMC9437719

[B54] Rastas P. (2017). Lep-MAP3: robust linkage mapping even for low-coverage whole genome sequencing data. Bioinformatics 33, 3726–3732. doi: 10.1093/bioinformatics/btx494 29036272

[B55] Rehman F. Gong H. Li Z. Zeng S. Yang T. Ai P.. (2020). Identification of fruit size associated quantitative trait loci featuring SLAF based high-density linkage map of goji berry (*Lycium* spp.). BMC Plant Bio. 20, 1–18. doi: 10.1186/s12870-020-02567-1 33059596 PMC7565837

[B56] Rehman F. Gong H. Bao Y. Zeng S. Huang H. Wang Y. (2022). CRISPR gene editing of major domestication traits accelerating breeding for solanaceae crops improvement. Plant Mol. Biol. 108 (3), 157–173. doi: 10.1007/s11103-021-01229-6 35032250

[B57] Ren Y. McGregor C. Zhang Y. Gong G. Zhang H. Guo S.. (2014). An integrated genetic map based on four mapping populations and quantitative trait loci associated with economically important traits in watermelon (*Citrullus lanatus*). BMC Plant Bio. 14, 1–11. doi: 10.1186/1471-2229-14-33 PMC3898567 24443961

[B58] Rodríguez-Leal D. Lemmon Z. H. Man J. Bartlett M. E. Lippman Z. B. (2017). Engineering quantitative trait variation for crop improvement by genome editing. Cell 171, 470–480. doi: 10.1016/j.cell.2017.08.030 28919077

[B59] Sandlin K. Prothro J. Heesacker A. Khalilian N. Okashah R. Xiang W.. (2012). Comparative mapping in watermelon [Citrullus lanatus (Thunb.) Matsum. et Nakai]. Theor. Appl. Genet. 125, 1603–1618. doi: 10.1007/s00122-012-1938-z 22875176

[B60] Sun L. Zhang Y. Cui H. Zhang L. Sha T. Wang C.. (2020). Linkage Mapping and comparative transcriptome analysis of firmness in watermelon (Citrullus lanatus). Front. Plant Sci. 11. doi: 10.3389/fpls.2020.00831 PMC7308538 32612625

[B61] Tanksley S. D. Fulton T. M. (2007). Dissecting quantitative trait variation—examples from the tomato. Euphytica. 154, 365–370. doi: 10.1007/s10681-006-9192-6

[B62] Tanksley S. D. Medina Filho H. Rick C. M. (1981). Using naturally occurring enzyme variation to detect and map genes controlling quantitative traits in an inter-specific backcross of tomato. Heredity 49, 11–25. doi: 10.1038/hdy.1982.61

[B63] Wang J. (2009). Inclusive composite interval mapping of quantitative trait genes. Acta Agron. Sin. 35, 239–245. doi: 10.3724/SP.J.1006.2009.00239

[B64] Wang K. Li M. Hakonarson H. (2021). ANNOVAR: functional annotation of genetic variants from next-generation sequencing data. Nucleic Acids Res. 38, e164. doi: 10.1093/nar/gkq603 PMC2938201 20601685

[B65] Wang S. Qiang Q. Xiang L. Fernie A. R. Yang J. (2023). Targeted approaches to improve tomato fruit taste. Horti. Res. 10, uhac229. doi: 10.1093/hr/uhac229 PMC9832879 36643745

[B66] Wang Y. Song F. Zhu J. Zhang S. Yang Y. Chen T.. (2017). GSA: genome sequence archive. Genom. Proteom. Bioinfor. 15, 14–18. doi: 10.1016/j.gpb.2017.01.001 PMC5339404 28387199

[B67] Wu X. Chen F. Zhao X. Pang C. Shi R. Liu C.. (2021). QTL mapping and GWAS reveal the genetic mechanism controlling soluble solids content in Brassica napus shoots. Foods 10, 2400. doi: 10.3390/foods10102400 34681449 PMC8535538

[B68] Wu X. Wu X. Wang Y. Wang B. Lu Z. Xu P.. (2019). Molecular genetic mapping of two complementary genes underpinning fruit bitterness in the bottle gourd (*Lagenaria siceraria* [Mol.] standl.). Front. Plant Sci. 10. doi: 10.3389/fpls.2019.01493 PMC6930244 31921223

[B69] Xu X. Bai G. (2015). Whole-genome resequencing: changing the paradigms of SNP detection, molecular mapping and gene discovery. Mol. Breed. 35, 1–11. doi: 10.1007/s11032-017-0664-2

[B70] Xu J. Driedonks N. Rutten M. J. Vriezen W. H. de Boer G. J. Rieu I. (2017). Mapping quantitative trait loci for heat tolerance of reproductive traits in tomato (*Solanum lycopersicum*). Mol. Breed. 37, 1–9. doi: 10.1007/s11032-017-0664-2 28479863 PMC5395597

[B71] Yao R. Heinrich M. Wang Z. Weckerle C. S. (2018). Quality control of goji (fruits of *Lycium barbarum* L. and *L. chinense* Mill.): A value chain analysis perspective. J. Ethnopharm. 224, 349–358. doi: 10.1016/j.jep.2018.06.010 29908314

[B72] Yuan H. Yuan H. Liu F. Zhao X. Z. Wu X. Y. Dong L. G.. (2017). Study on bitter taste character of wild *Lycium barbarum* L. @ in China. J. Plant Genet. Resour. 18 (5), 991–1000. doi: 10.13430/j.cnki.jpgr.2017.05.023

[B73] Yuan H. Yuan H. Peng J. Yin M. Chen Y. Zhao X. Z.. (2022). Survey of wild bitter wolfberry germplasm in Ningxia autonomous region and Shaanxi province, China and analysis of the chemical constituents. J. Plant Genet. Resour. 23, 1400–1413. doi: 10.13430/j.cnki.jpgr.20220620002

[B74] Yue Y. Wei A. Zhao J. H. Li Y. L. Fan Y. F. Chen J. H.. (2022). Constructing the wolfberry (*Lycium* spp.) genetic linkage map using AFLP and SSR markers. J. Integ. Agric. 21, 131–138. doi: 10.1016/S2095-3119(21)63610-9

[B75] Zao S. Huang T. Qin K. Dai G.L. (2018). Study on storability of different wolfberry fresh fruit strains. Ningxia J. Agric. Fores. Sci. Tech. 59 (6), 2,17–18,21. doi: 10.3969/j.issn.1002-204x.2018.06.008

[B76] Zhang Q. Chen W. Zhao J. Xi W. (2016). Functional constituents and antioxidant activities of eight Chinese native goji genotypes. Food Chem. 200, 230–236. doi: 10.1016/j.foodchem.2016.01.046 26830583

[B77] Zhang L. Li H. Li Z. Wang J. (2008). Interactions between markers can be caused by the dominance effect of quantitative trait loci. Genetics. 180, 1177–1190. doi: 10.1534/genetics.108.092122 18780741 PMC2567366

[B78] Zhang L. Meng L. Wu W. Wang J. (2015). GACD: Integrated software for genetic analysis in clonal F1 and double cross populations. J. Heredity. 106, 741–744. doi: 10.1093/jhered/esv080 26503825

[B79] Zhang Z. Zhao W. Xiao J. Bao Y. He S. Zhang G.. (2020). Database resources of the national genomics data center. Nucleic Acids Res. 48, D24–D33. doi: 10.1093/nar/gkz913 31702008 PMC7145560

[B80] Zhao J. Li H. Xu Y. Yin Y. Huang T. Zhang B.. (2021). A consensus and saturated genetic map provides insight into genome anchoring, synteny of Solanaceae and leaf-and fruit-related QTLs in wolfberry (*Lycium* Linn.). BMC Plant Biol. 21, 1–13. doi: 10.1186/s12870-021-03115-1 34303361 PMC8306383

[B81] Zhao J. Xu Y. Li H. Yin Y. An W. Li Y.. (2019). A SNP-based high-density genetic map of leaf and fruit related quantitative trait loci in wolfberry (*Lycium* Linn.). Front. Plant Sci. 10. doi: 10.3389/fpls.2019.00977 PMC6693522 31440266

[B82] Zhou J. Liu Q. Tian R. Chen H. Wang J. Yang Y.. (2024). A co-located QTL for seven spike architecture-related traits shows promising breeding use potential in common wheat (*Triticum aestivum* L.). Theor. Appl. Genet. 137, 1–5. doi: 10.1007/s00122-023-04536-2 38267732

