# Supplementary figures and images for: An ultra-dense linkage map identified quantitative trait loci corresponding to fruit quality- and size-related traits in red goji berry
Source: Front Plant Sci. 2024 Sep 4;15:1390936. doi: 10.3389/fpls.2024.1390936 (PMC11408189; doi:10.3389/fpls.2024.1390936)

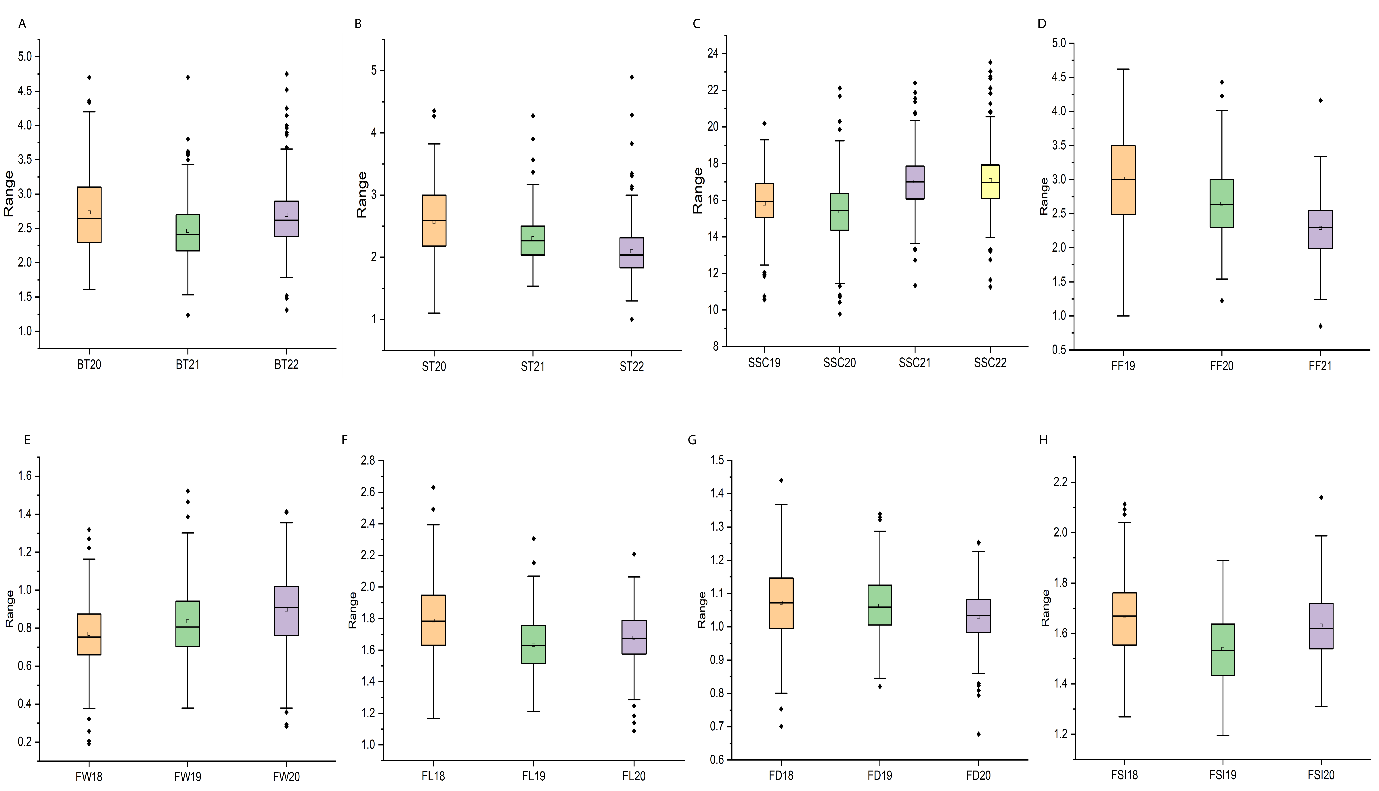


FIGURE S1

**
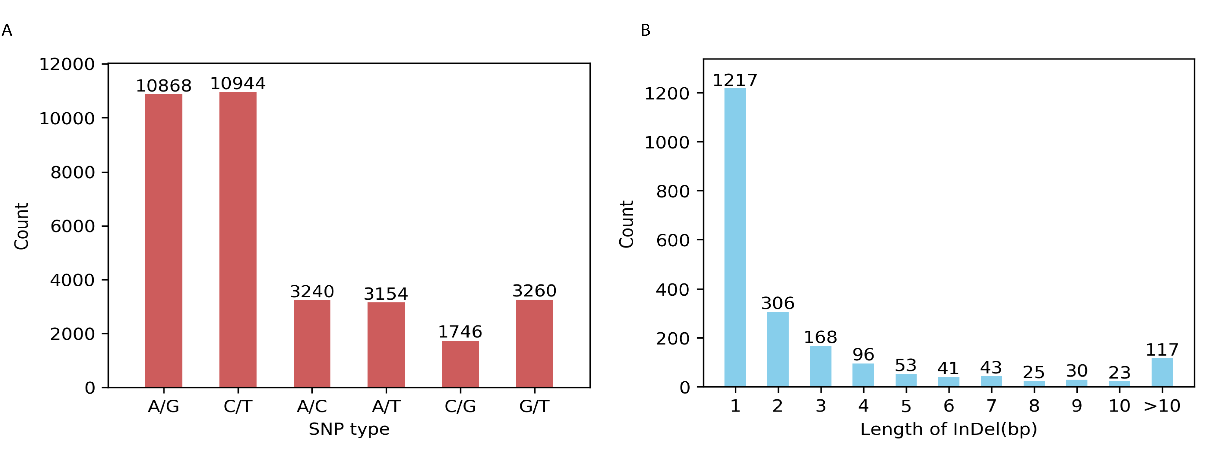
**

FIGURE S2

**
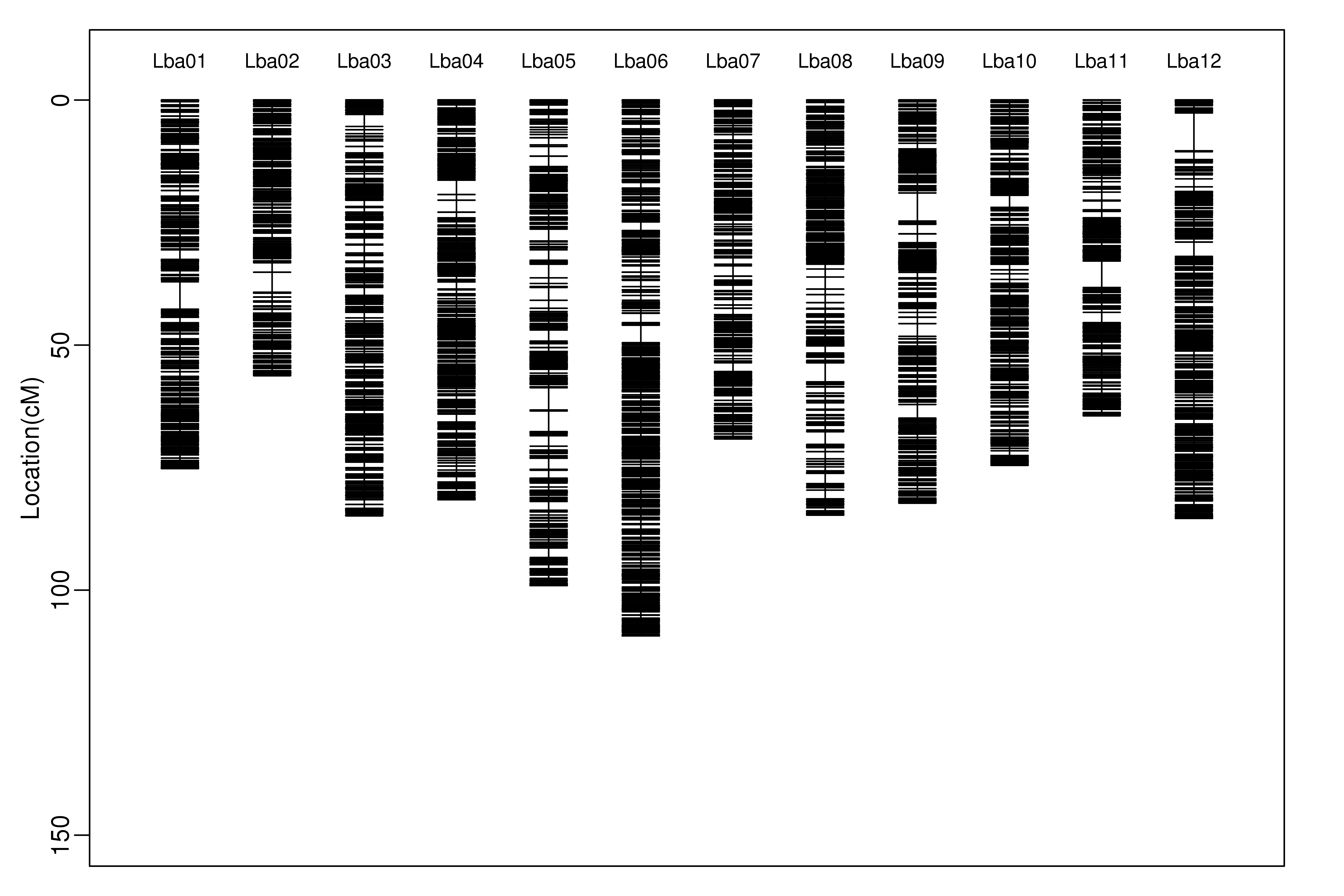
**

FIGURE S3


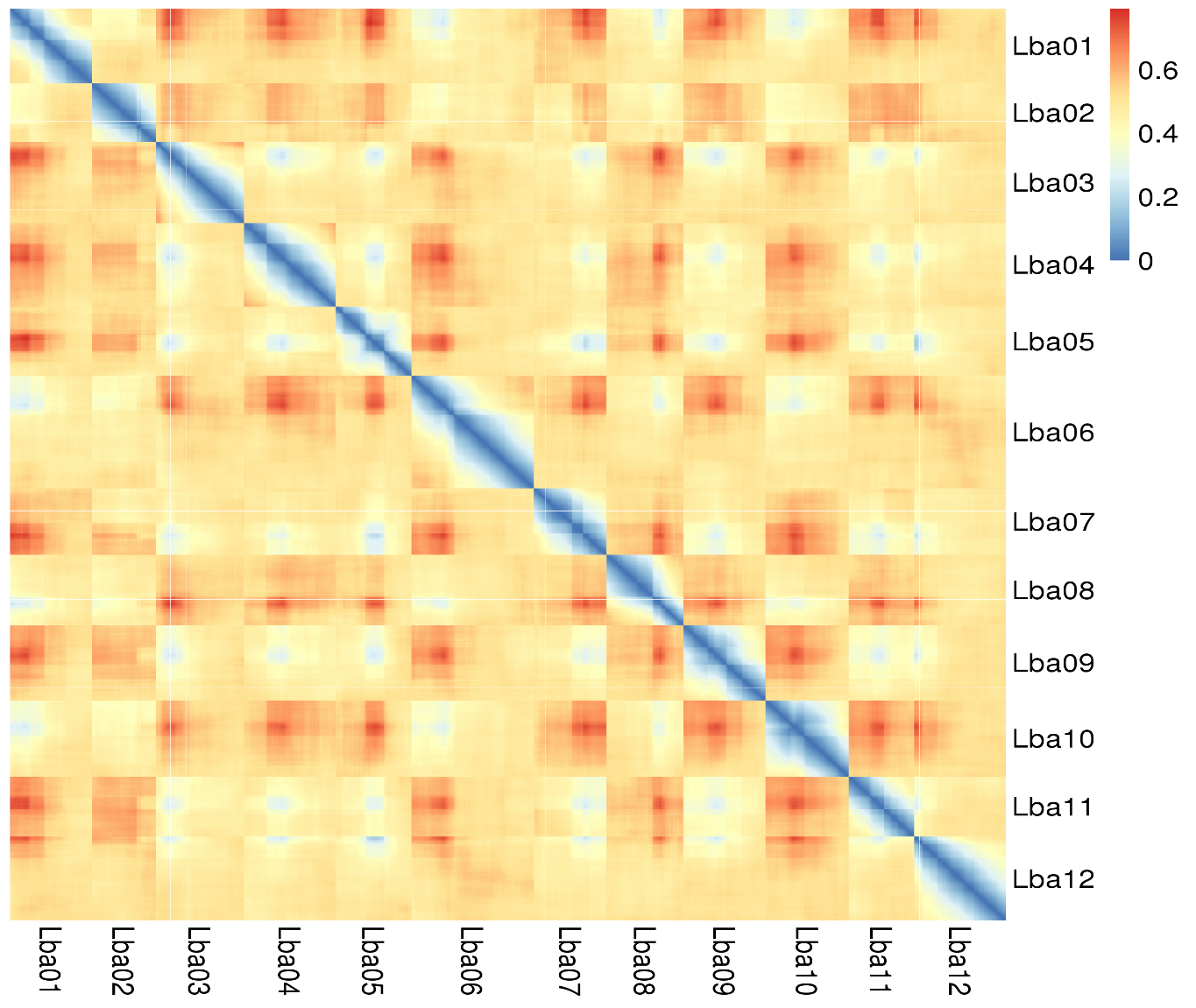

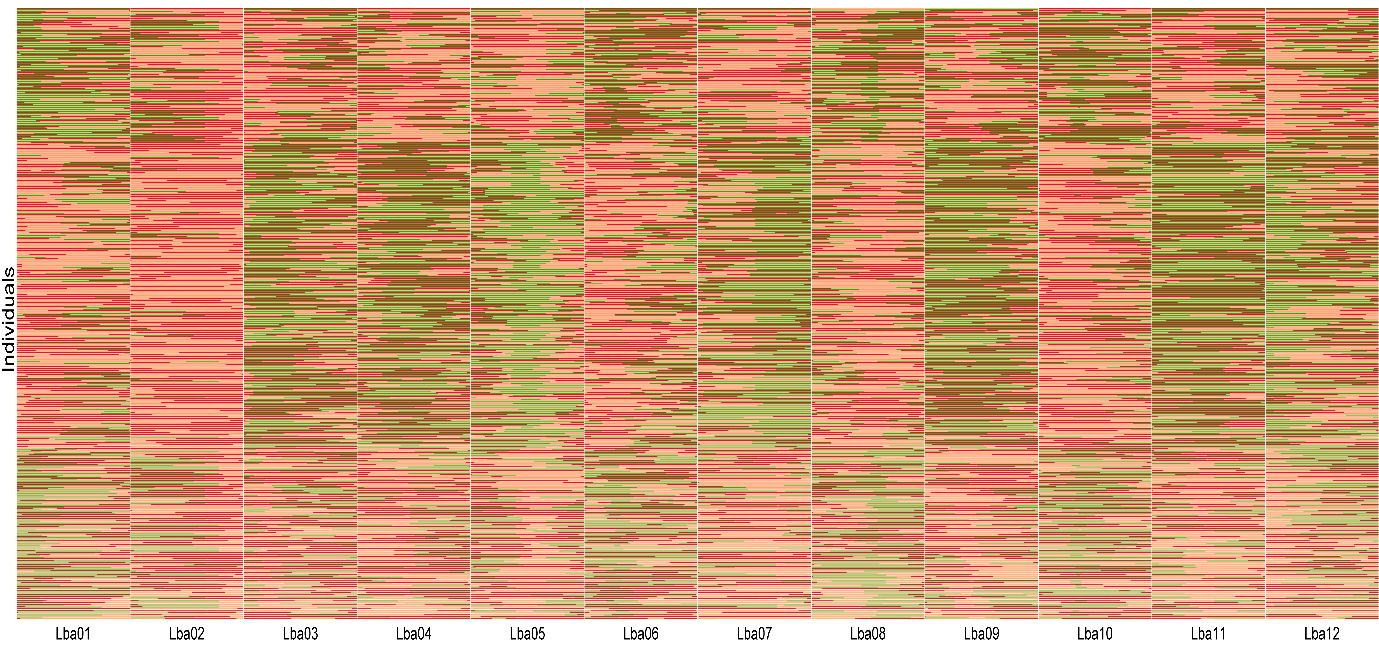
 FIGURE S4

FIGURE S5


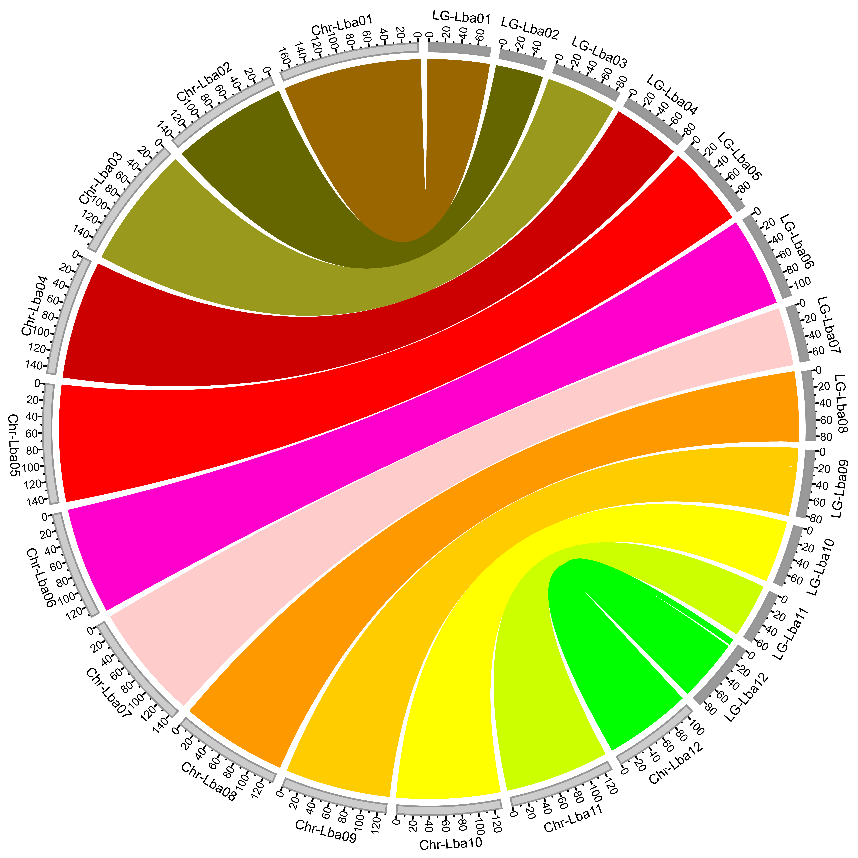


FIGURE S6

Supplement: Supplementary Figure 1 — Box chart of 305 F1 individuals for physio-chemical and morphological traits based on three or four individual years. (A–H) Box chart diagram of 305 F1 individuals BT, ST, SSC, FF, FW, FL, FD, and FSI based on three or four individual years. Each x-axis represents the trait in different years, and the y-axis shows the frequency of ranges corresponding to the value on the x-axis. In each box chart, the lower and upper lines represent the first and third quartiles, respectively, and the middle line represents the median. BT20, BT21, BT22 bitter taste (2020, 2021, 2022), ST20, ST21, ST22 sweet taste (2020, 2021, 2022), SSC19, SSC20, SSC21, SSC22, soluble solid content (°brix percentage) (2019, 2020, 2021, 2022); FF19, FF20, FF21, fruit firmness (2019, 2020, 2021); FW18, FW19, FW20, fruit weight (2018, 2019, 2020); FL18, FL19, FL20, fruit length (2018, 2019, 2020); FD18, FD19, FD20, fruit diameter (2018, 2019, 2020); FSI18, FSI19, FSI20, fruit shape index (2018, 2019, 2020). [file DataSheet1.docx]

Lba01

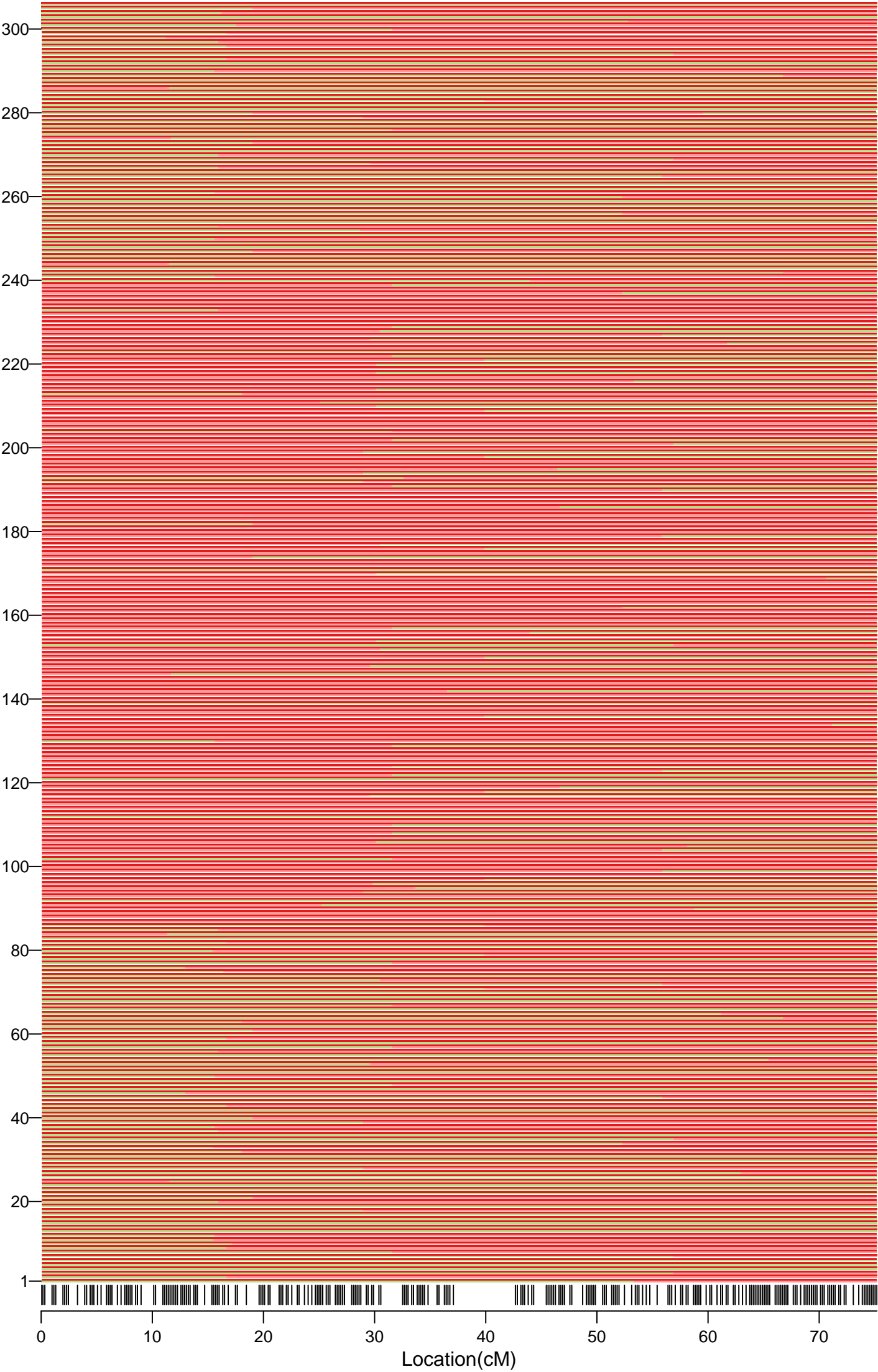

Lba02

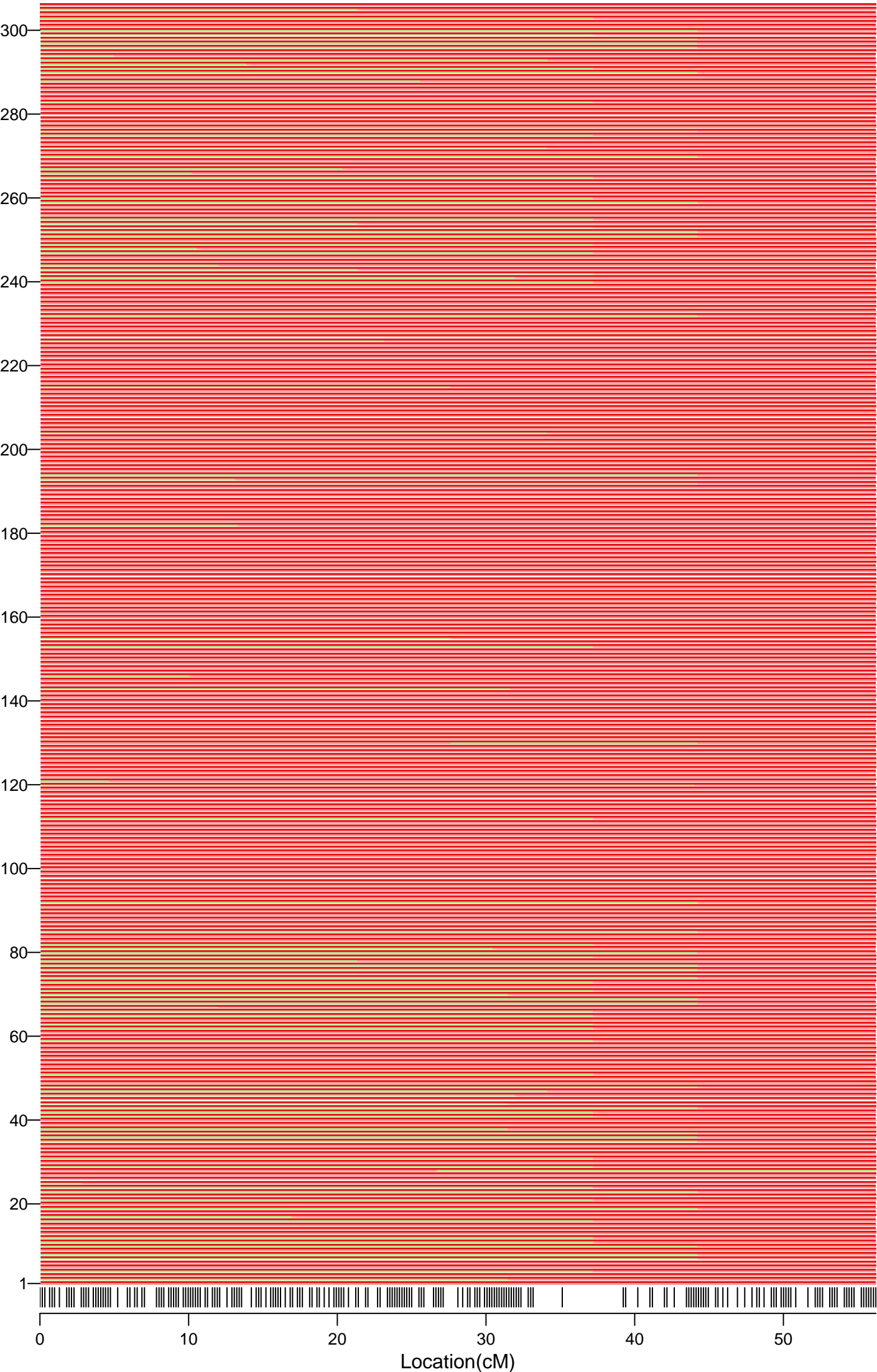

Lba03

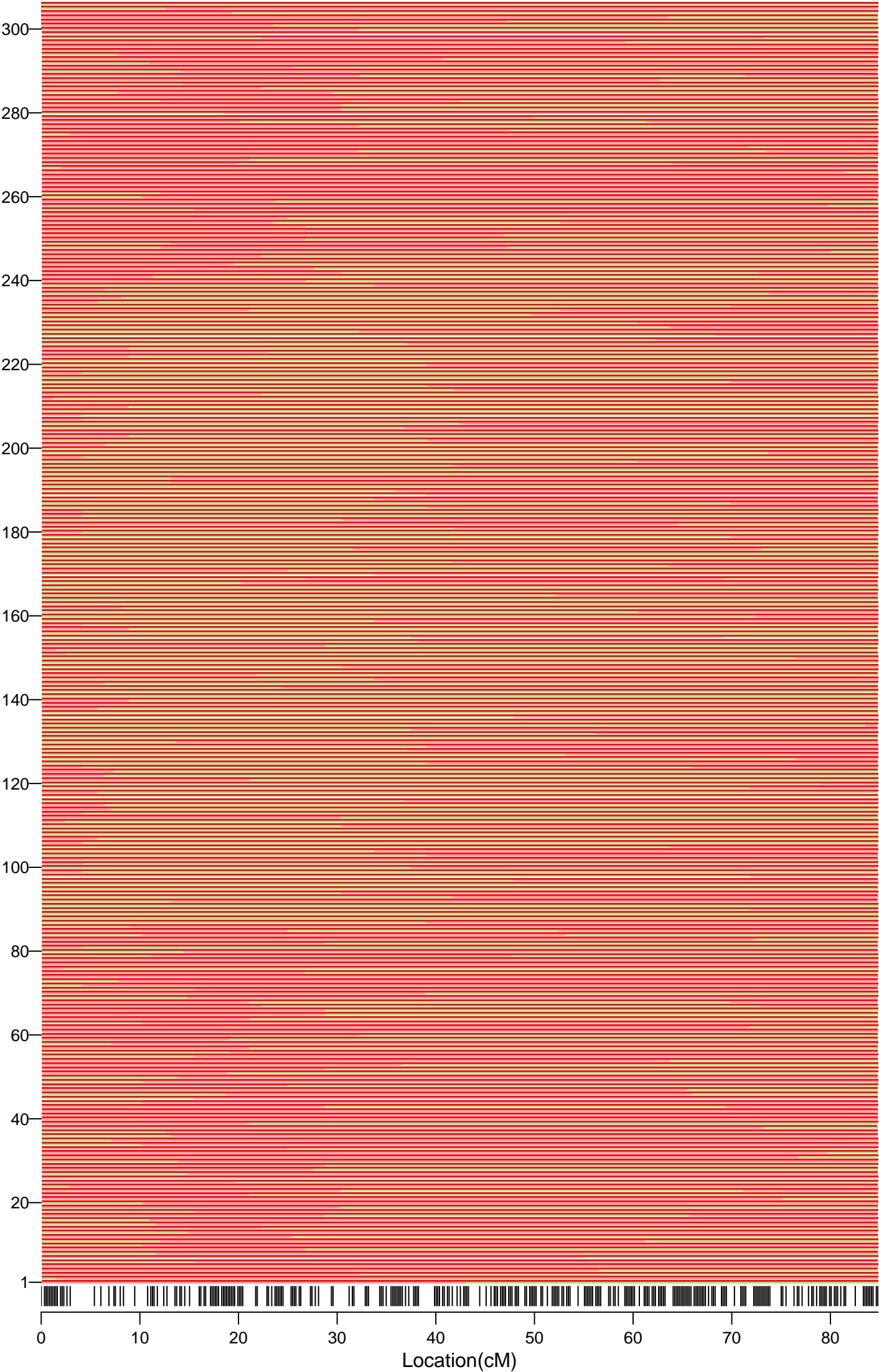

Lba04

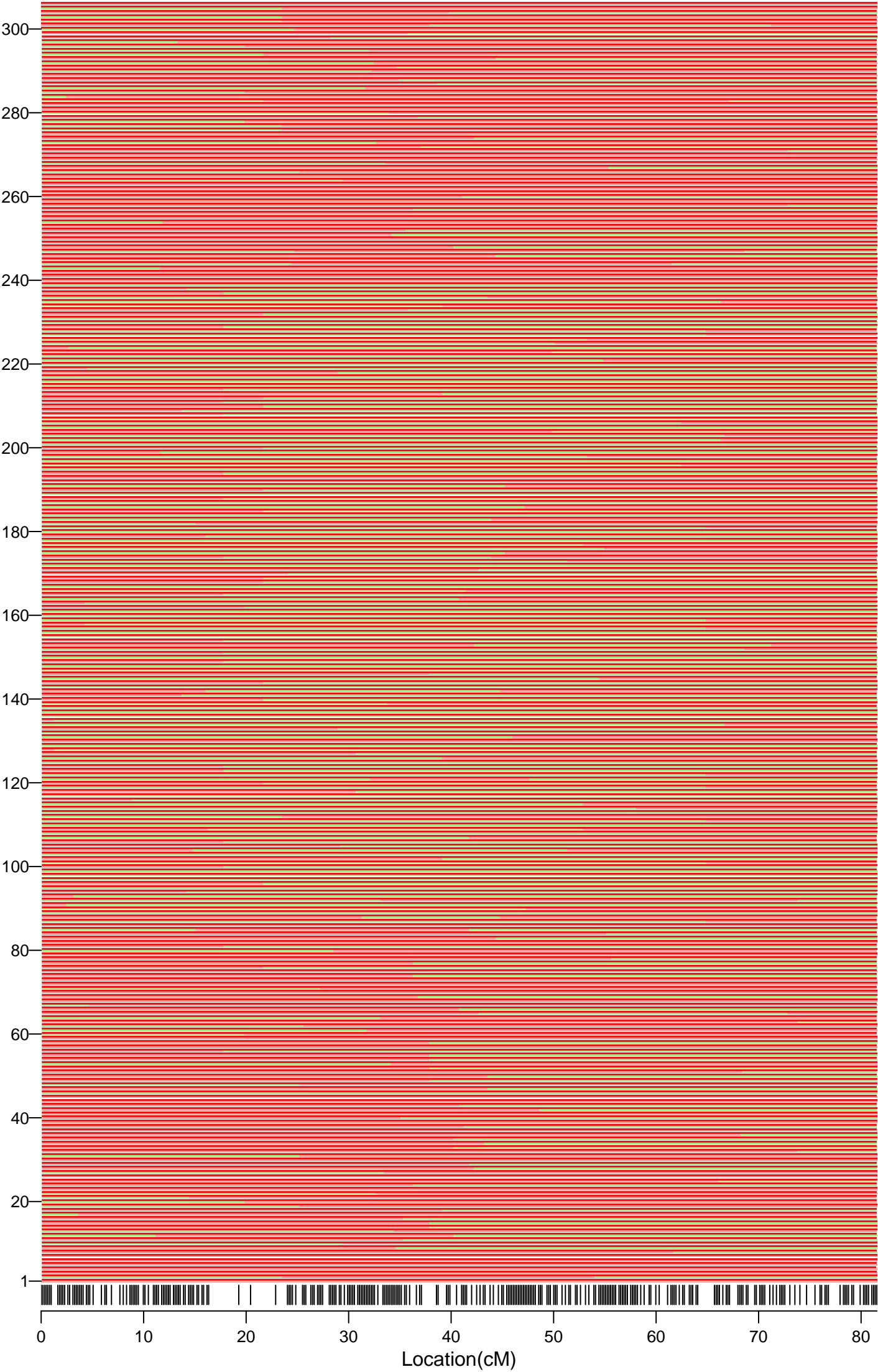

Lba05

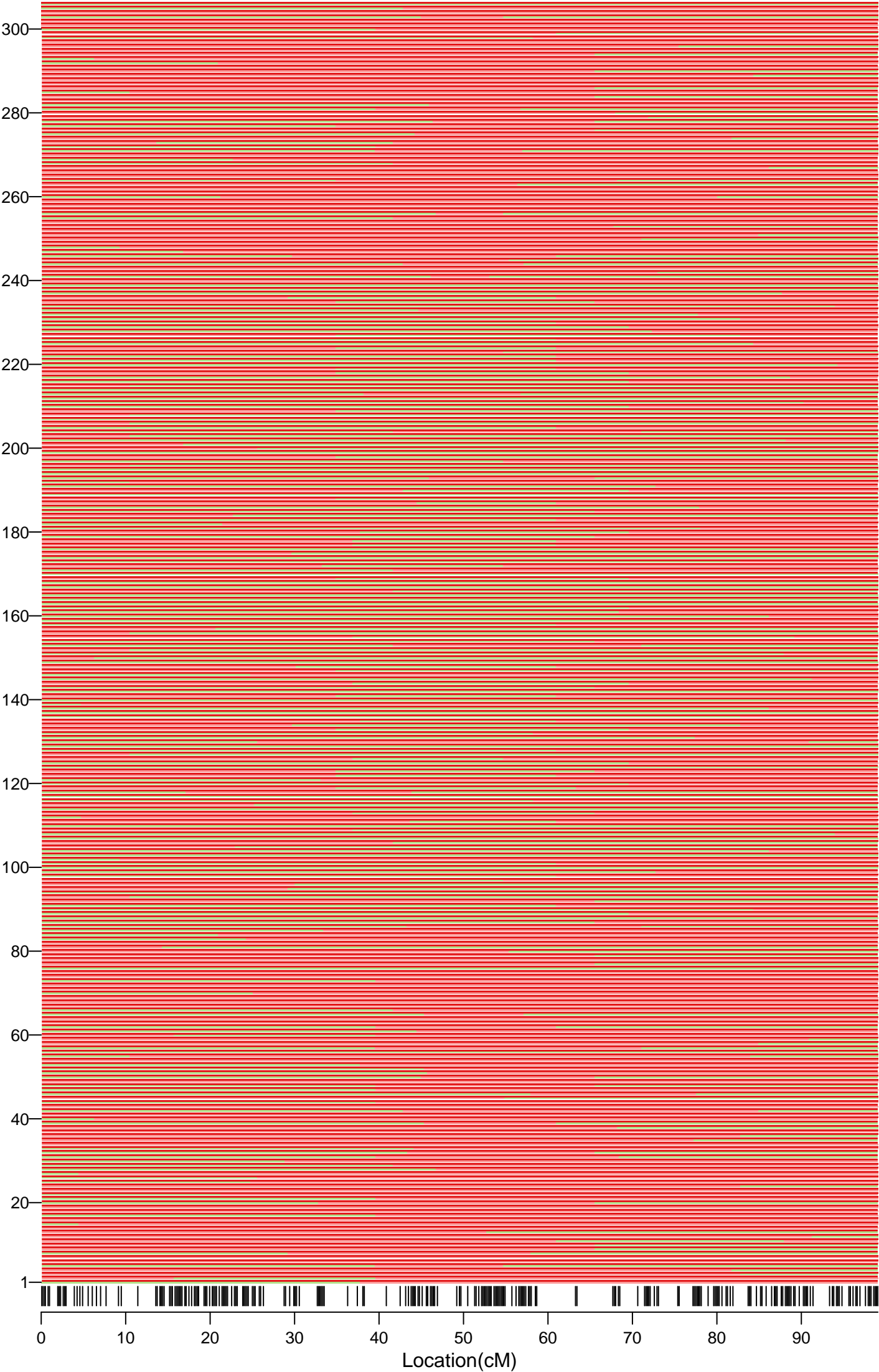

Lba06

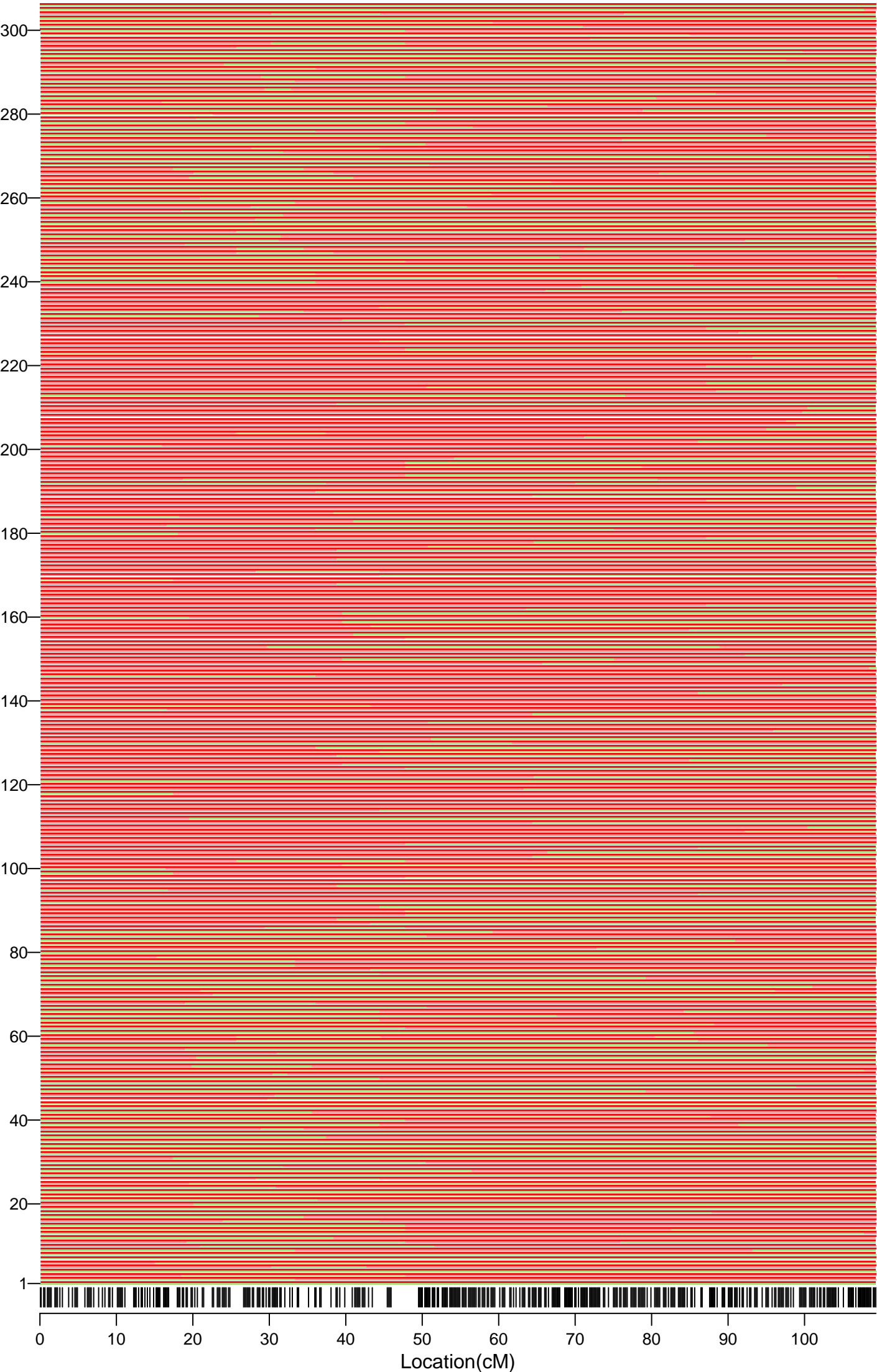

Lba07

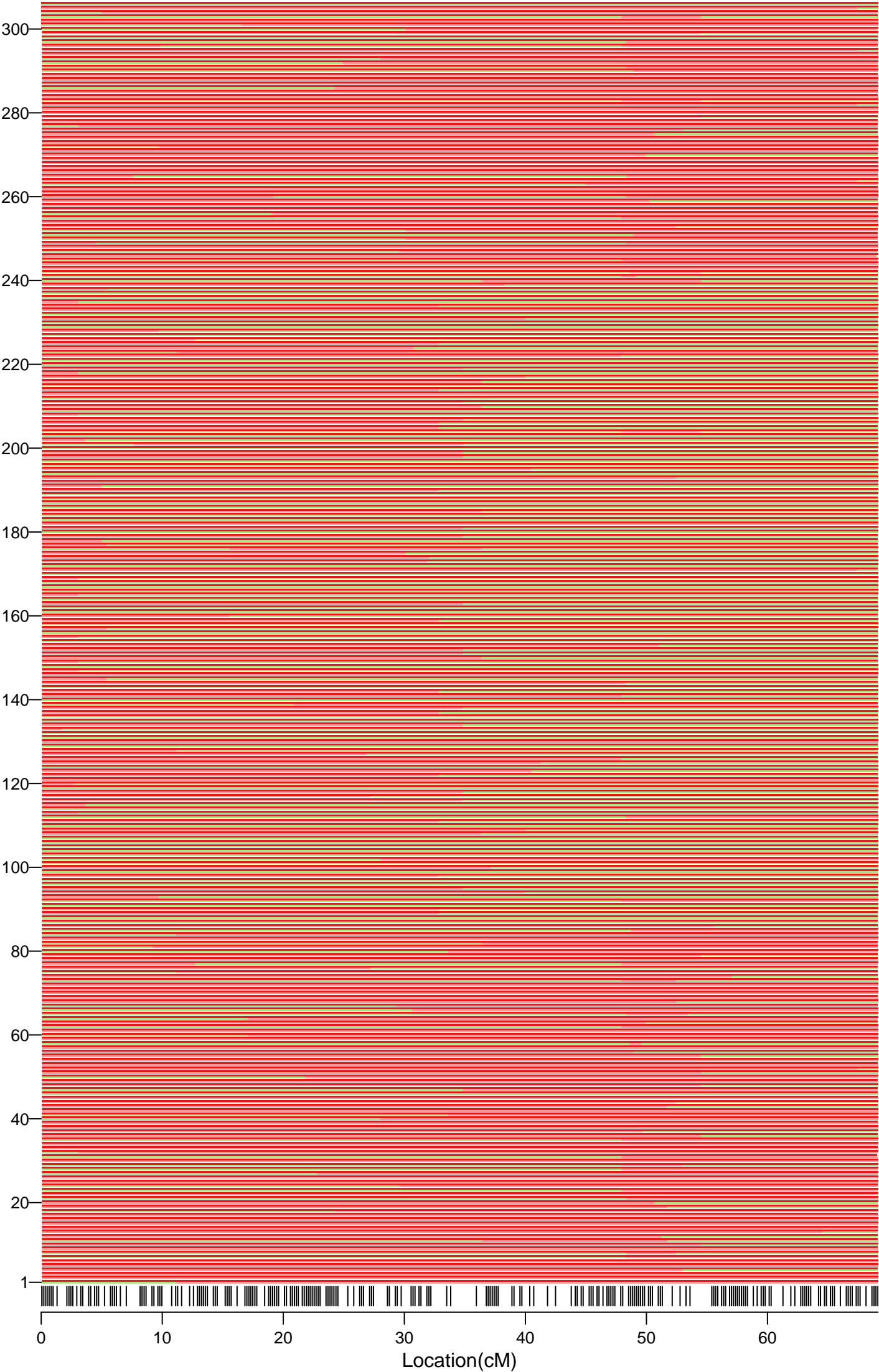

Lba08

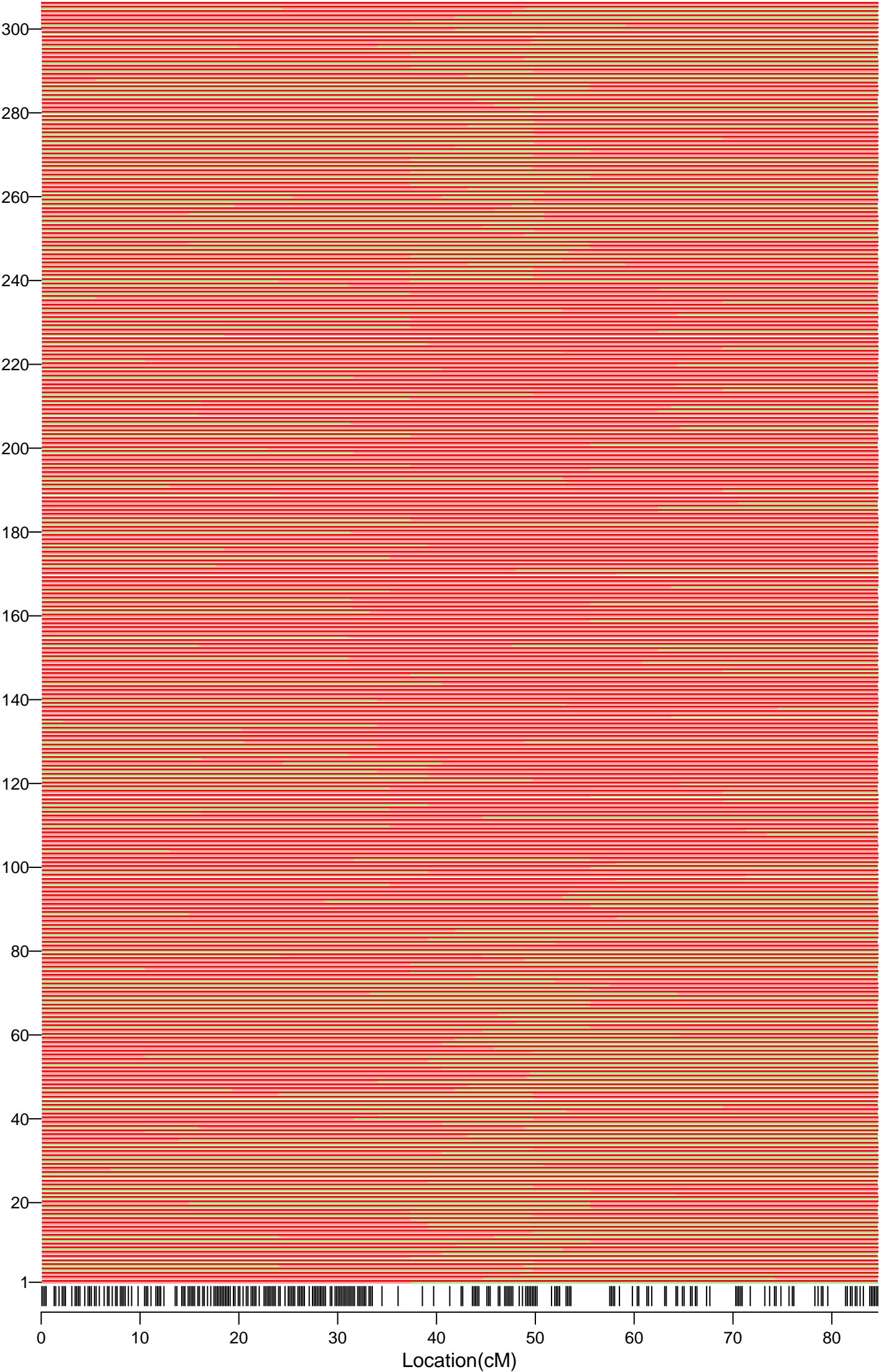

Lba09

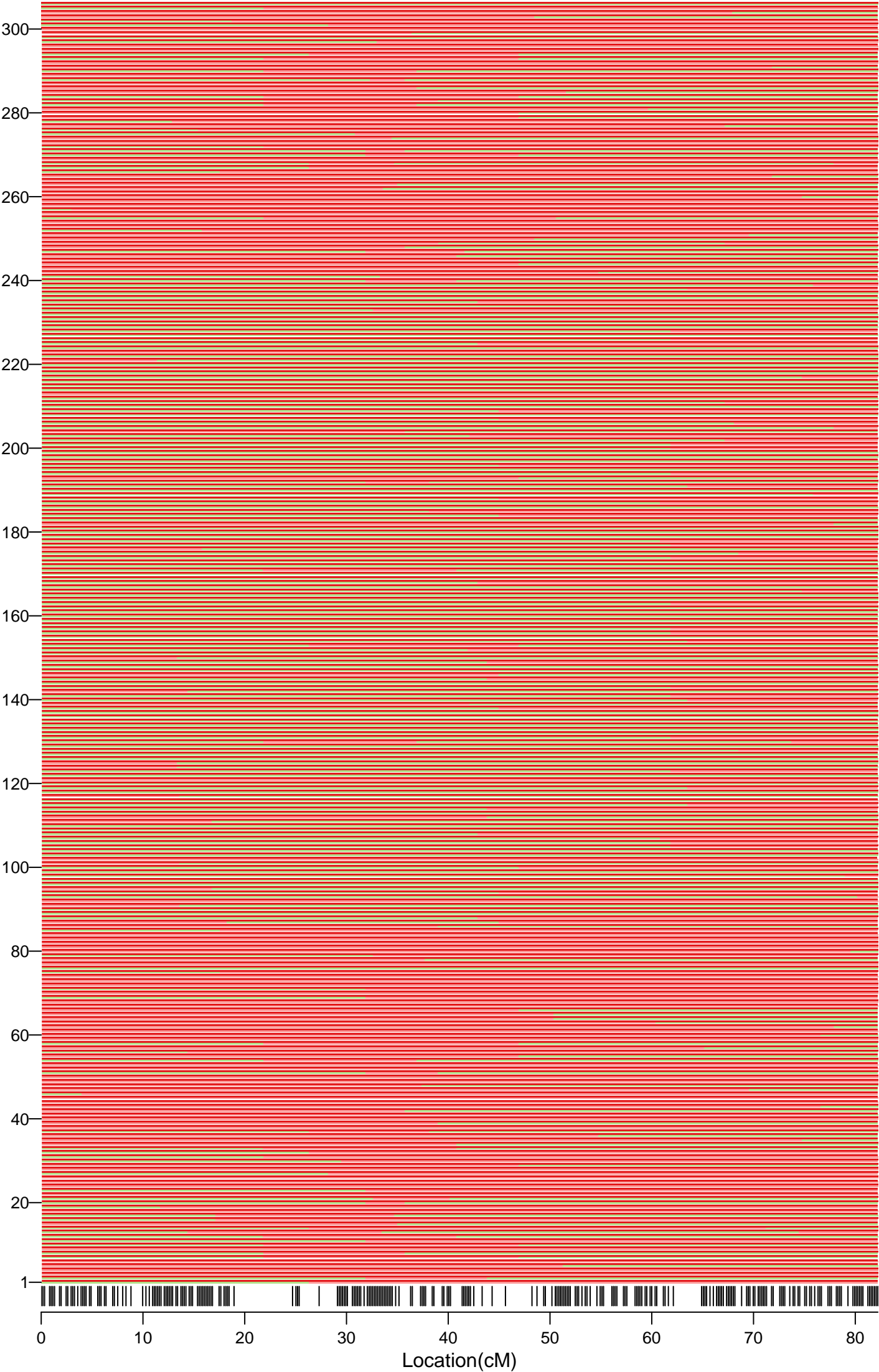

Lba10

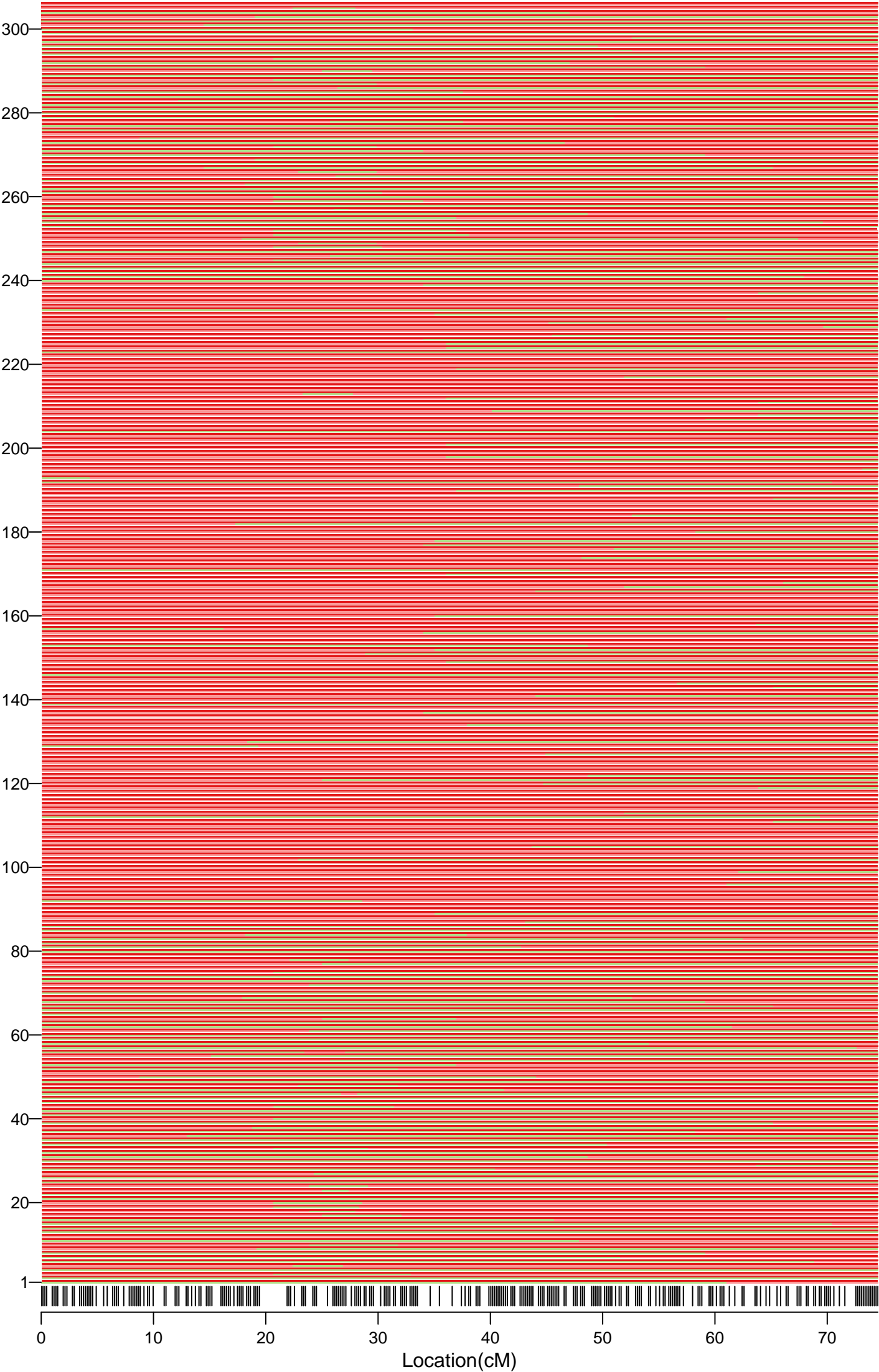

Lba11

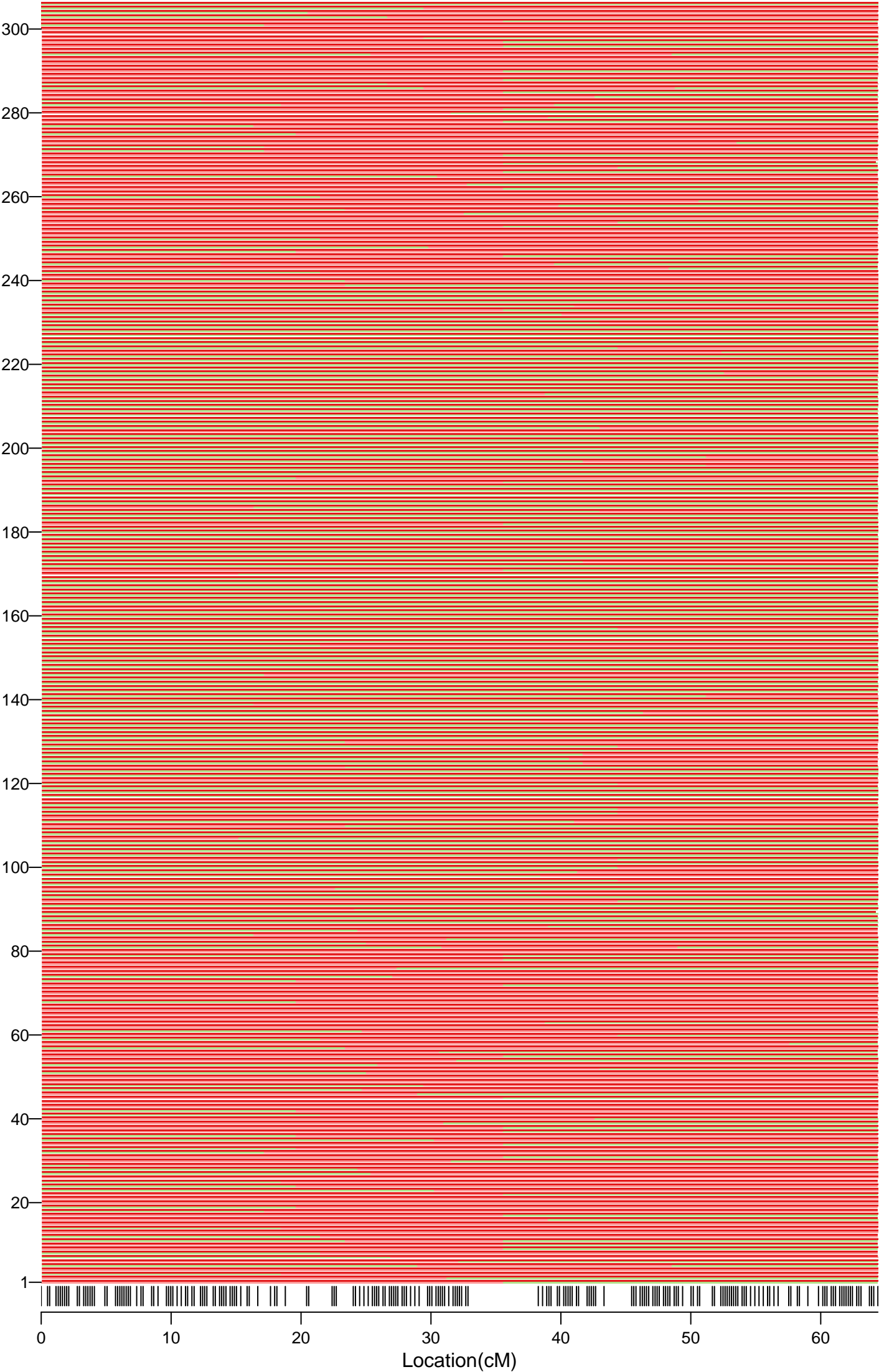

Lba12

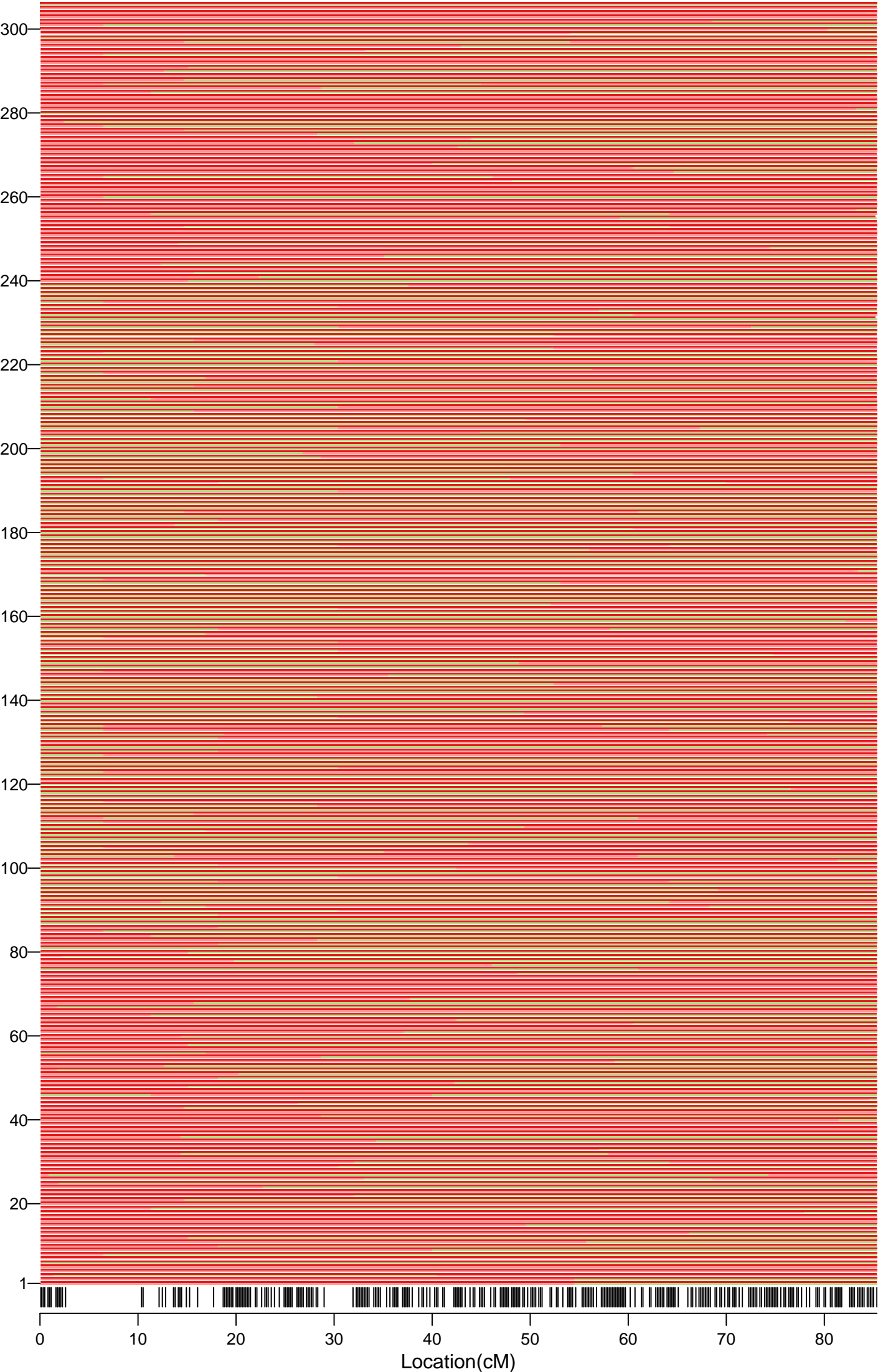

Supplement: Supplementary file 2 [file DataSheet1.pdf]

Lba01

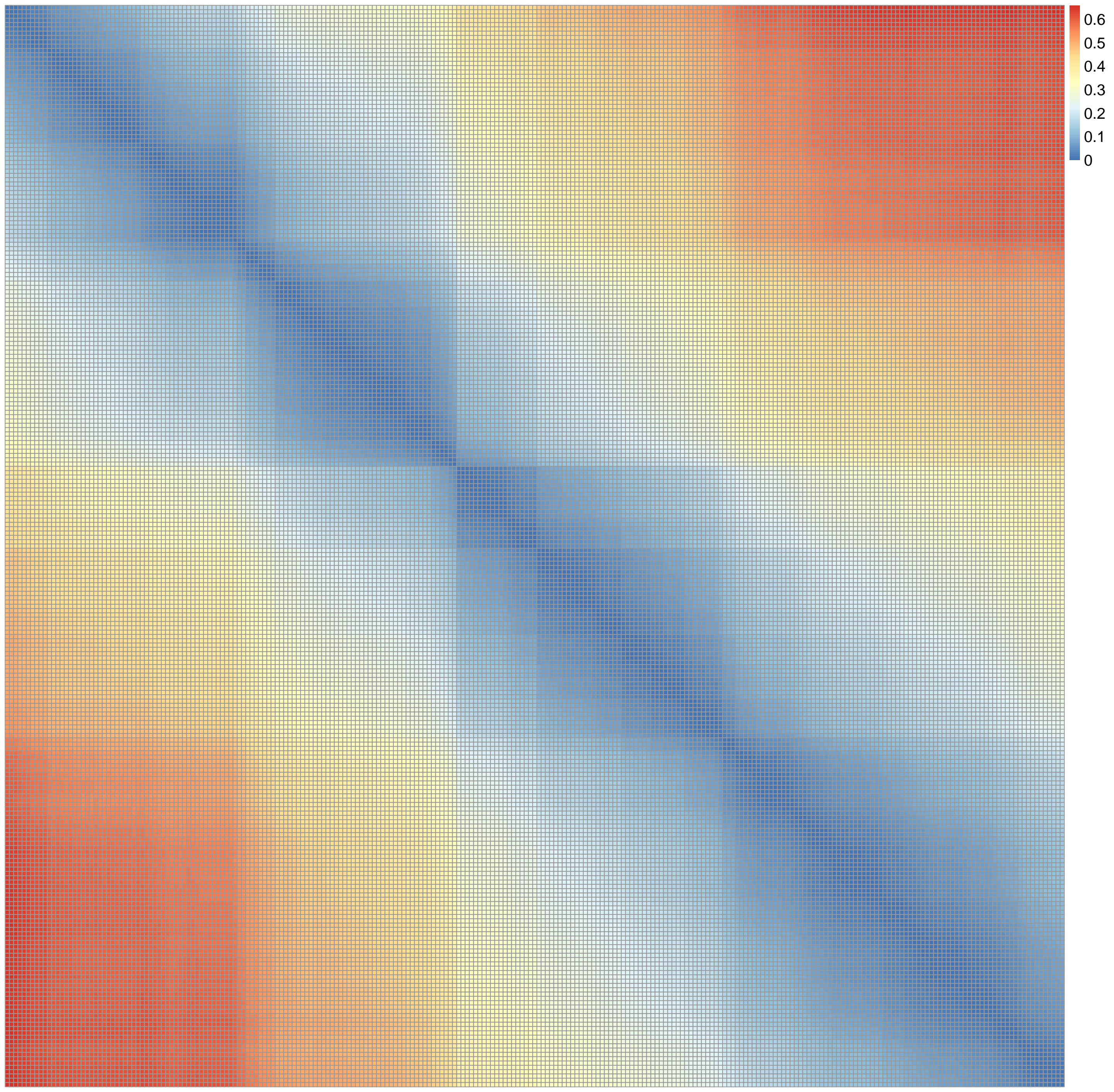

Supplement: Supplementary file 3 [file DataSheet2.pdf]

Lba02

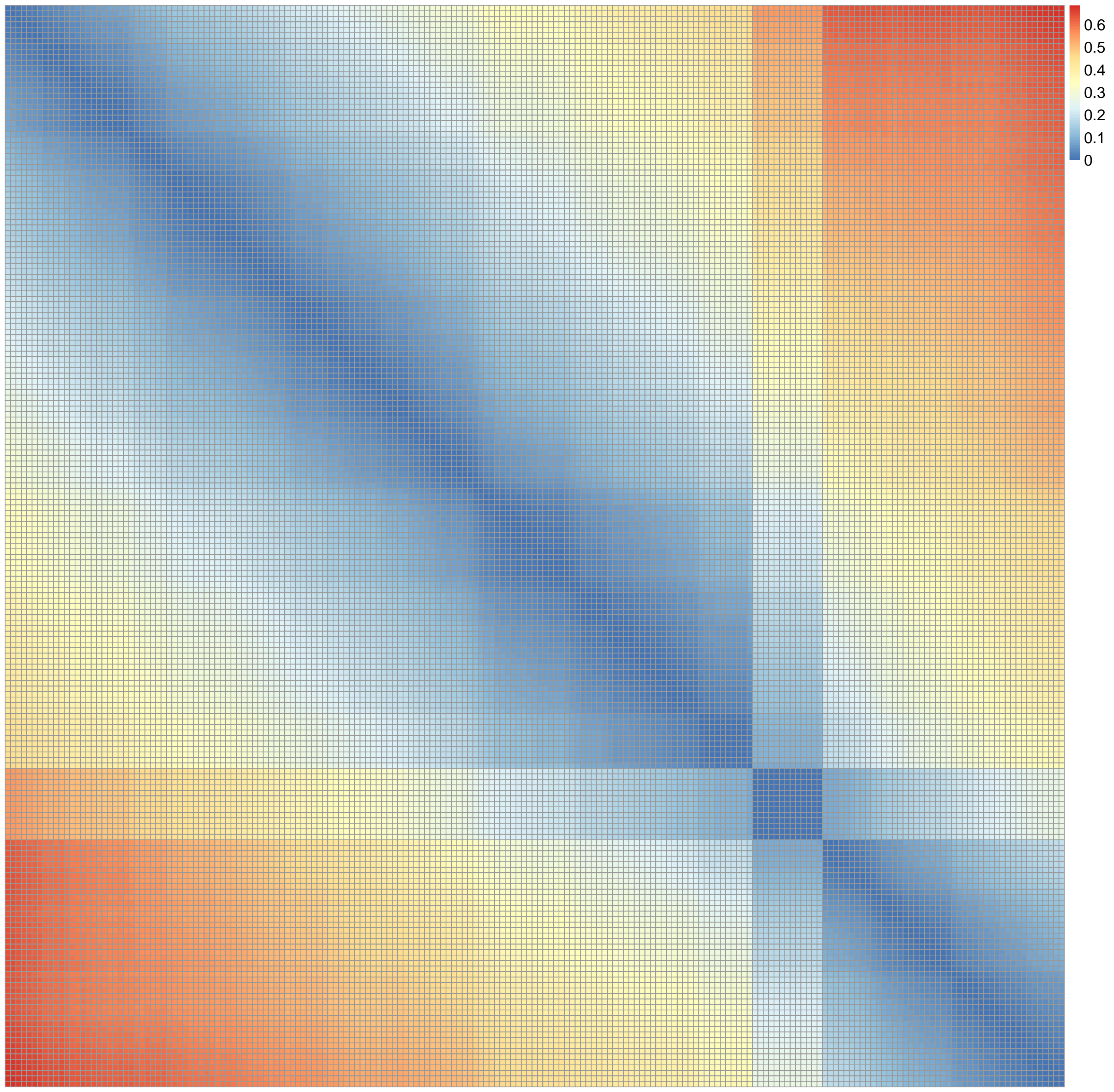

Supplement: Supplementary file 4 [file DataSheet3.pdf]

Lba03

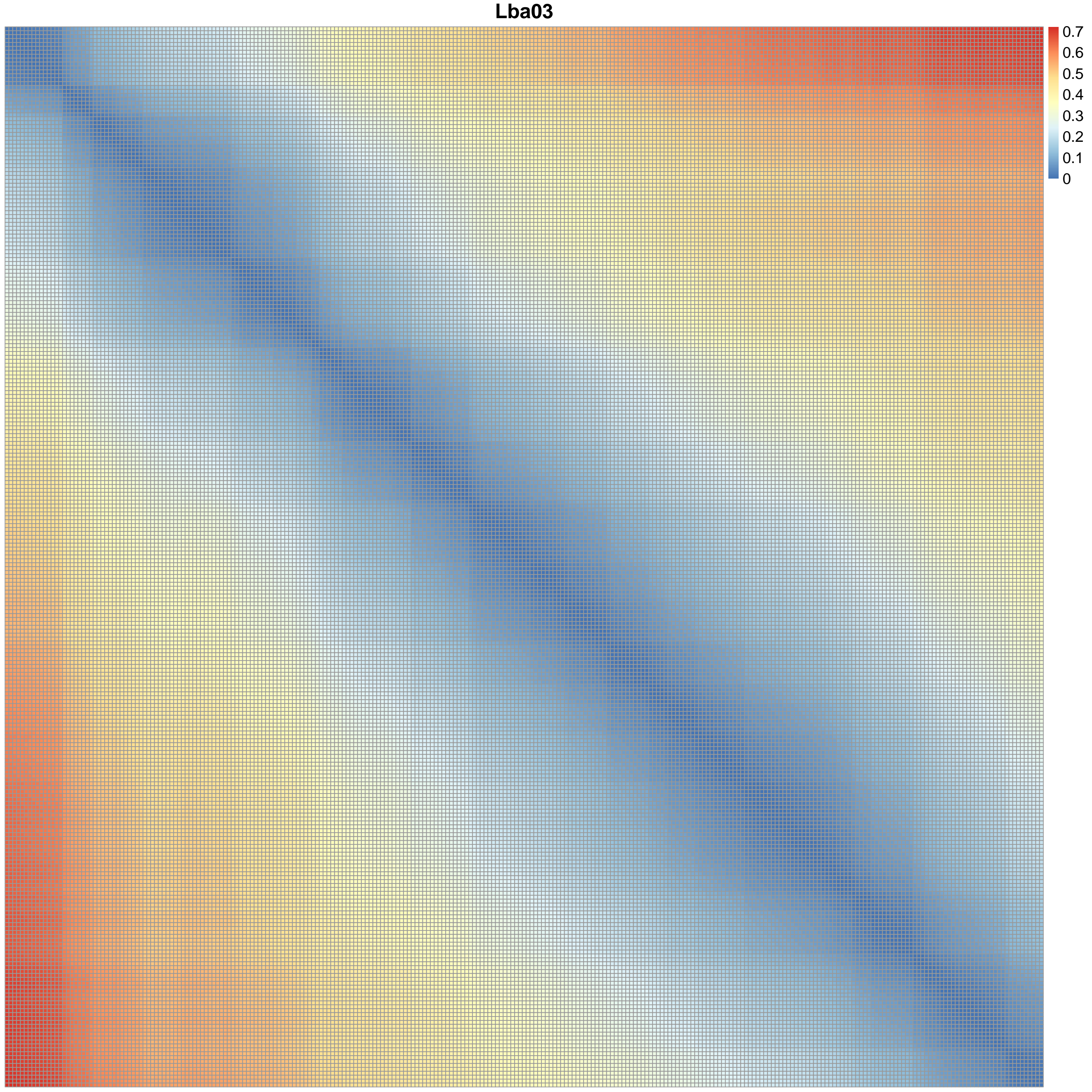

Supplement: Supplementary file 5 [file DataSheet4.pdf]

Lba04

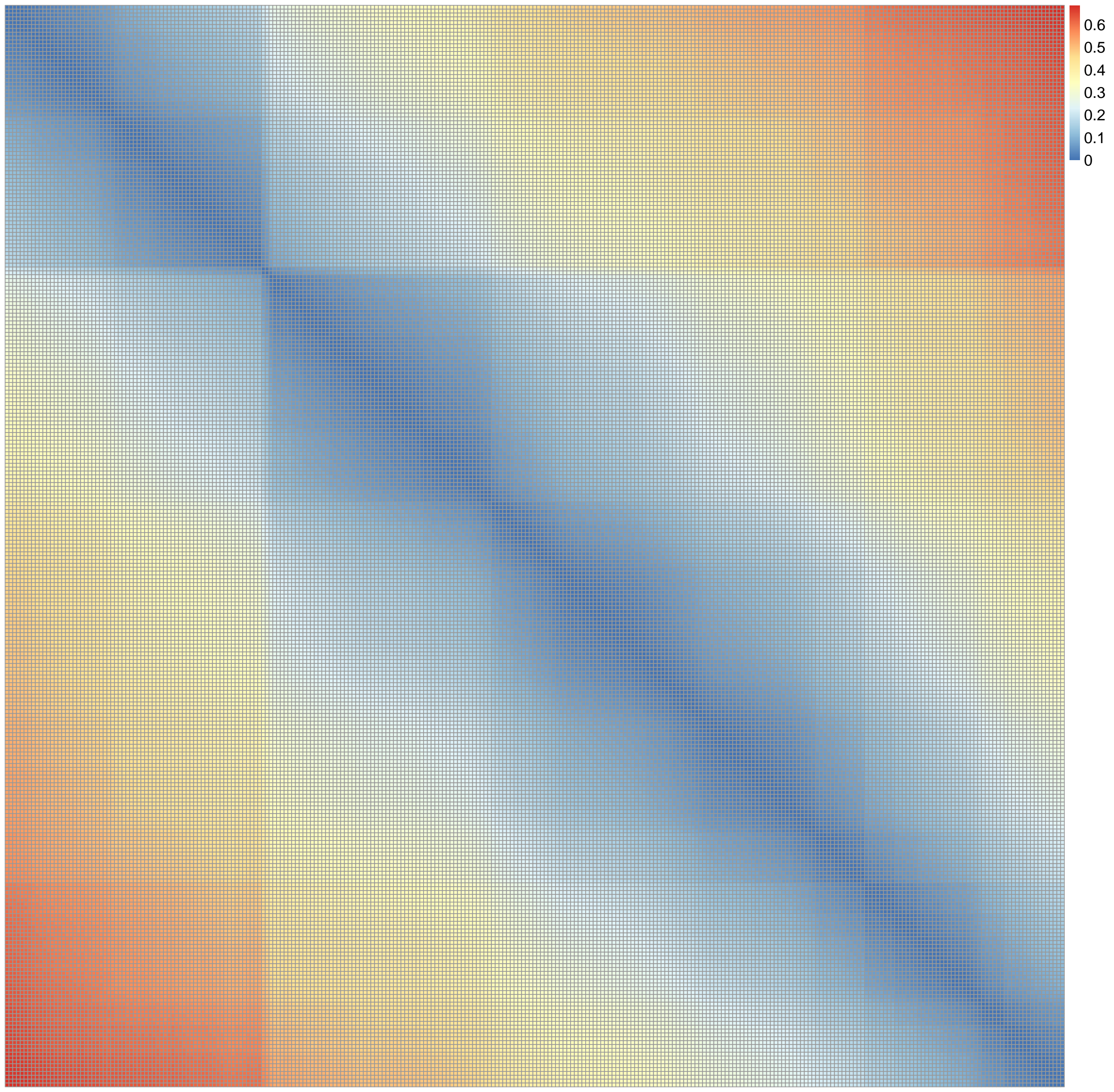

Supplement: Supplementary file 6 [file DataSheet5.pdf]

Lba05

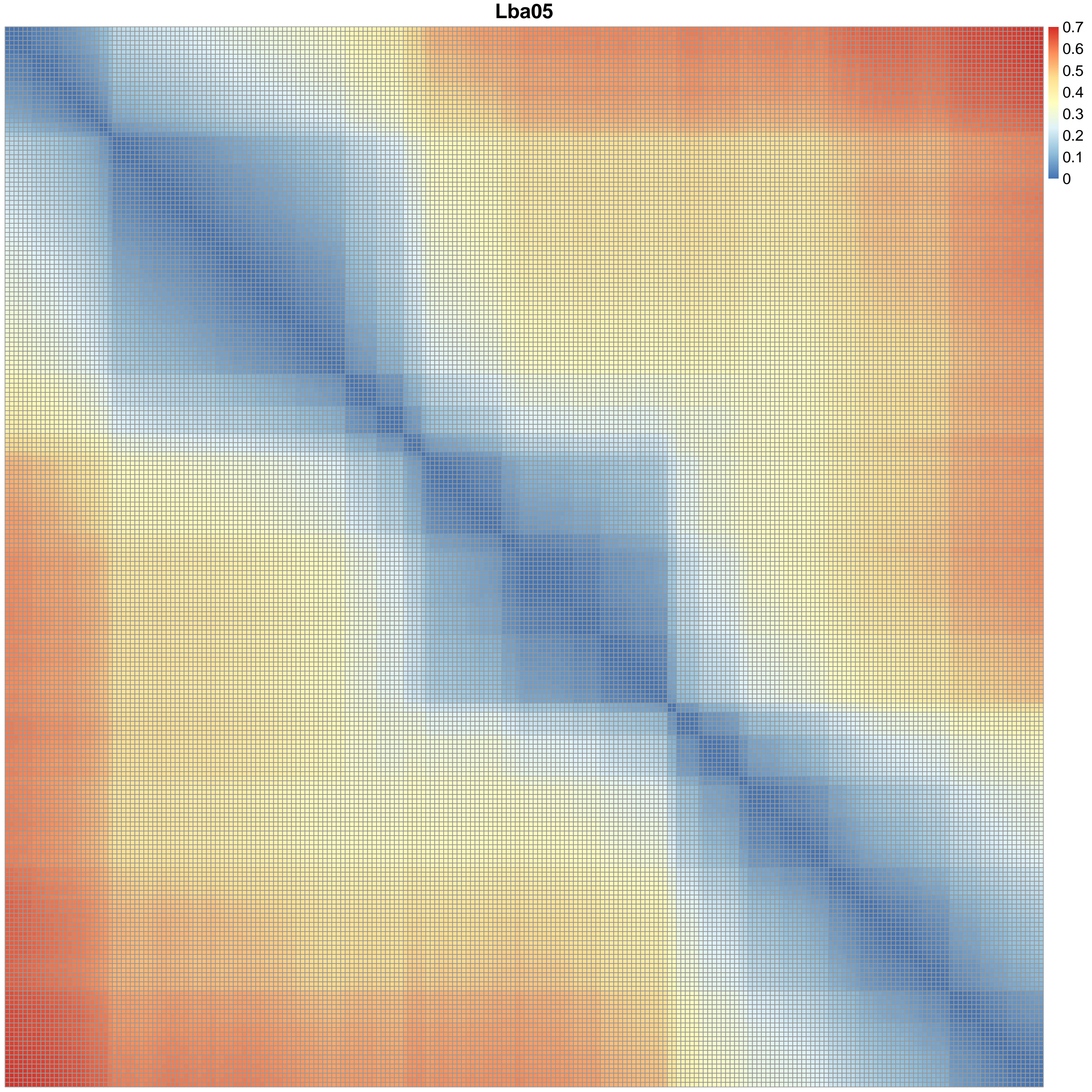

Supplement: Supplementary file 7 [file DataSheet6.pdf]

Lba06

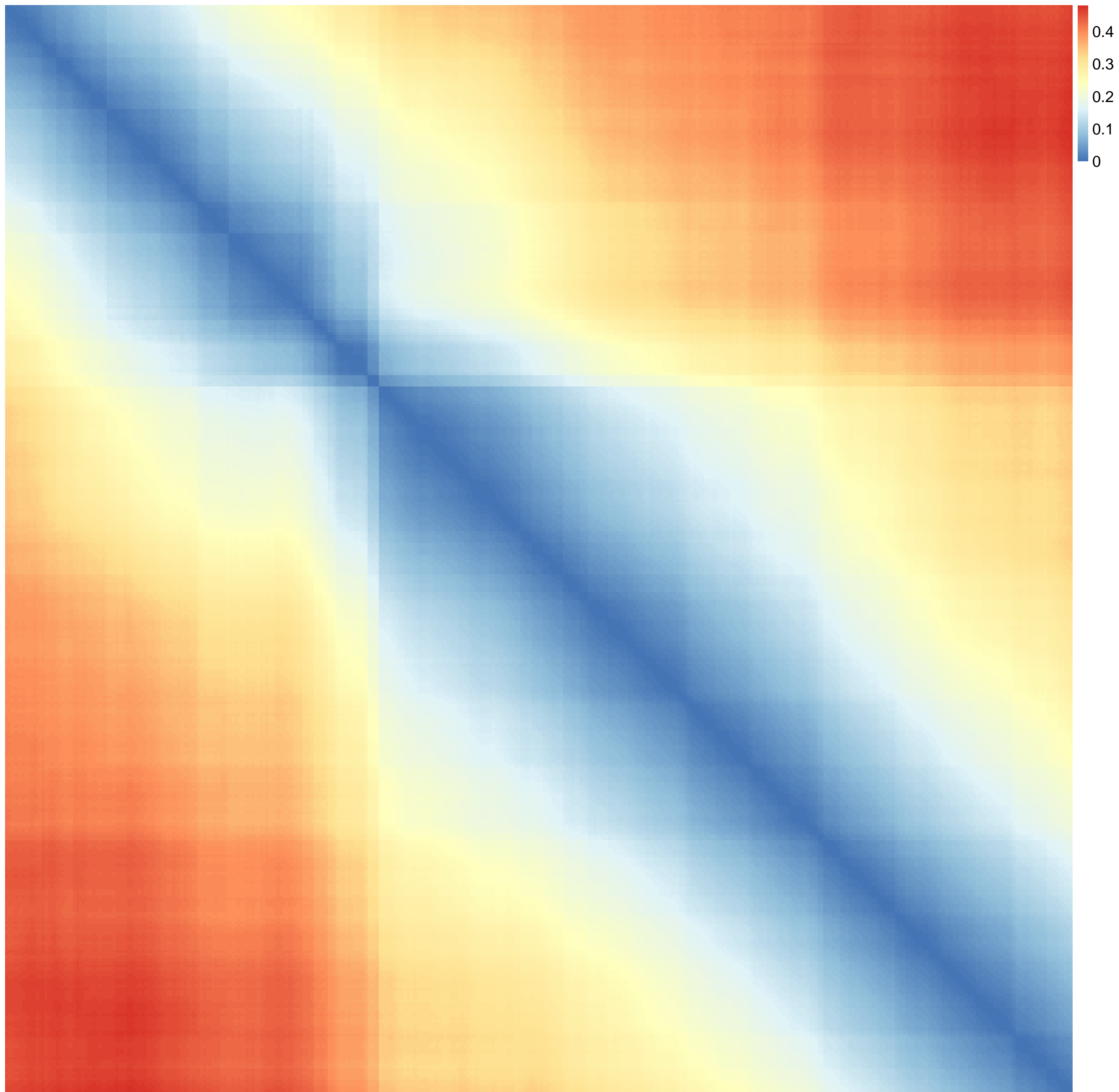

Supplement: Supplementary file 8 [file DataSheet7.pdf]

Lba07

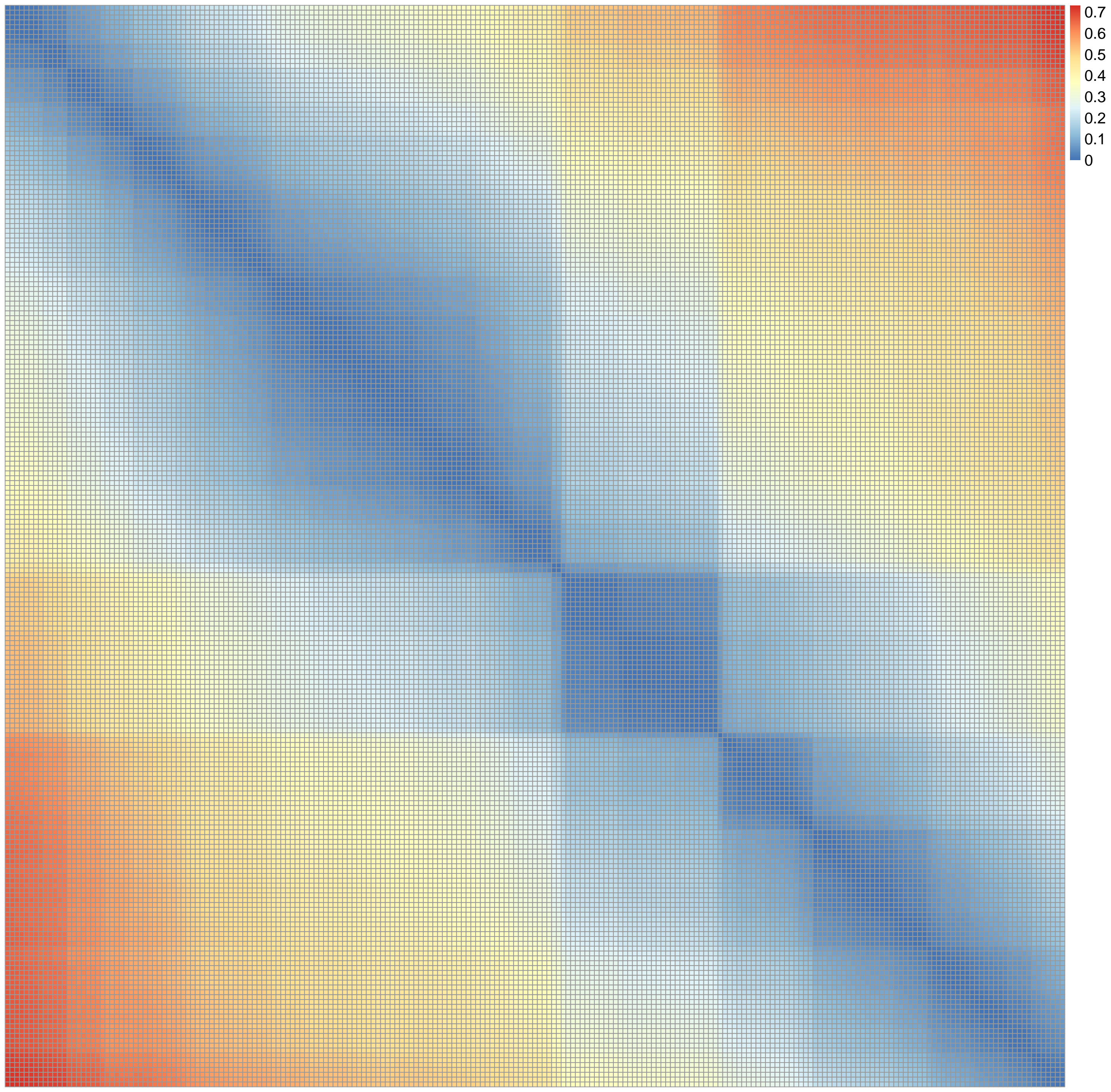

Supplement: Supplementary file 9 [file DataSheet8.pdf]

Lba08

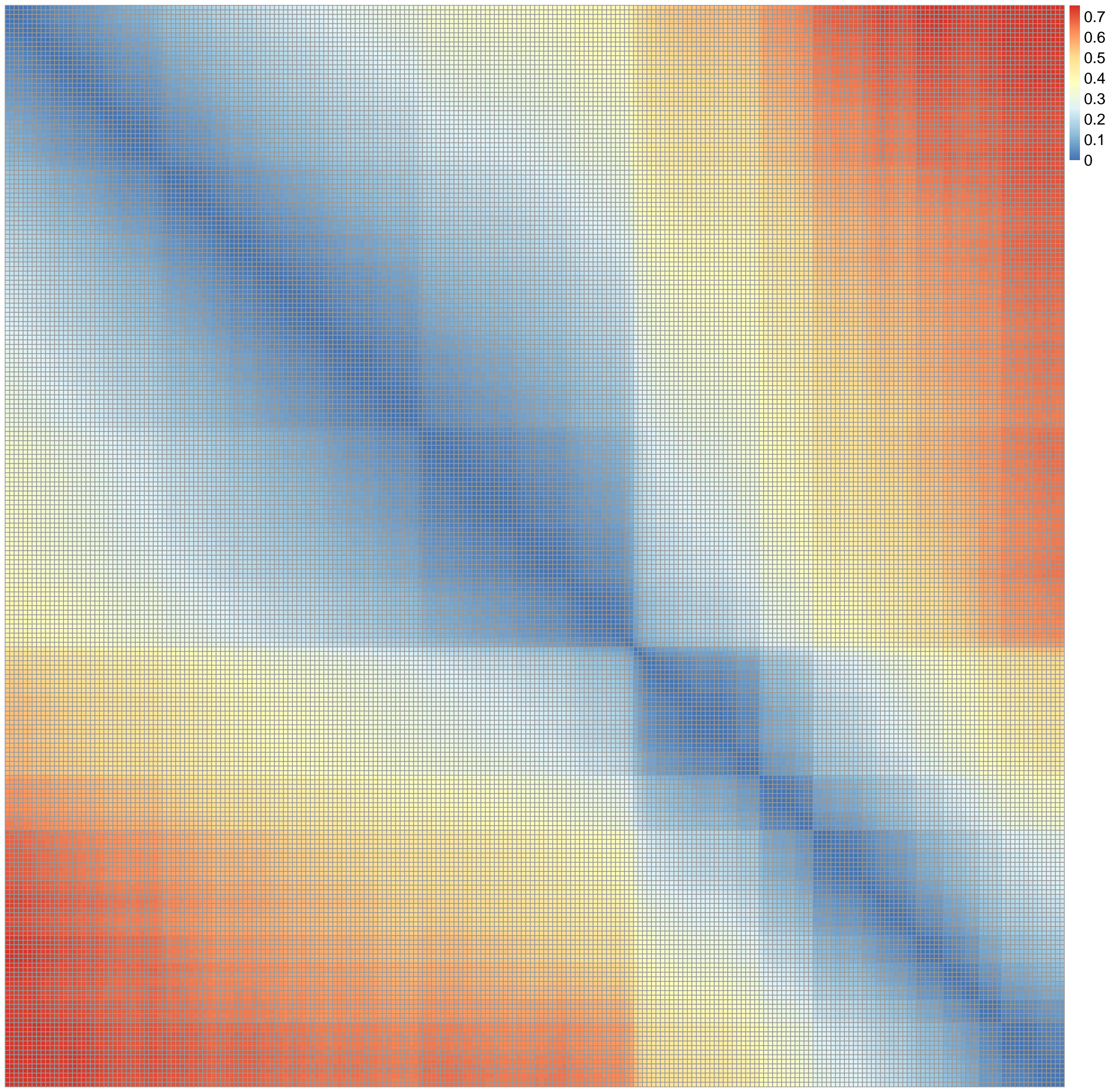

Supplement: Supplementary file 10 [file DataSheet9.pdf]

Lba09

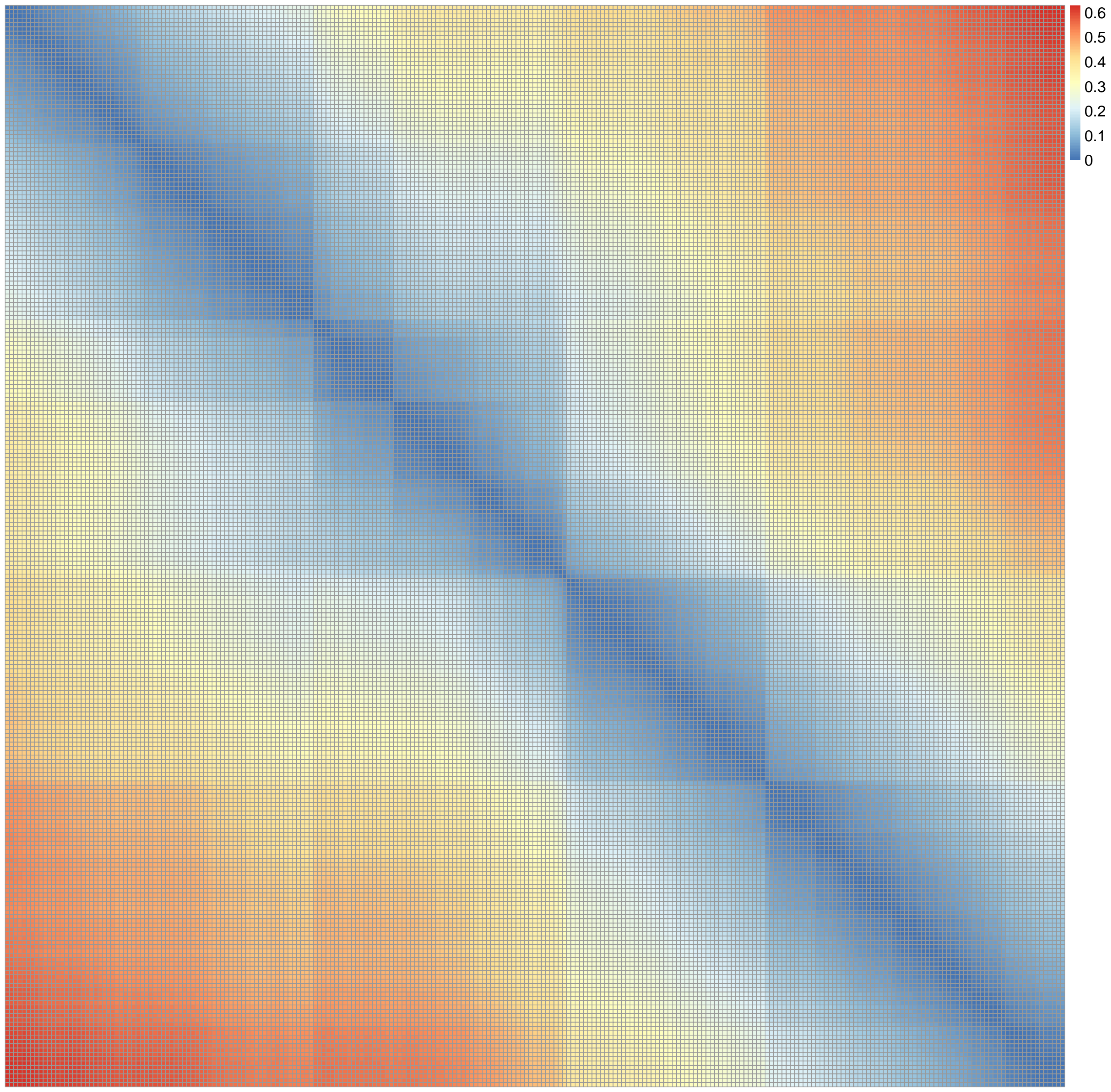

Supplement: Supplementary file 11 [file DataSheet10.pdf]

Lba10

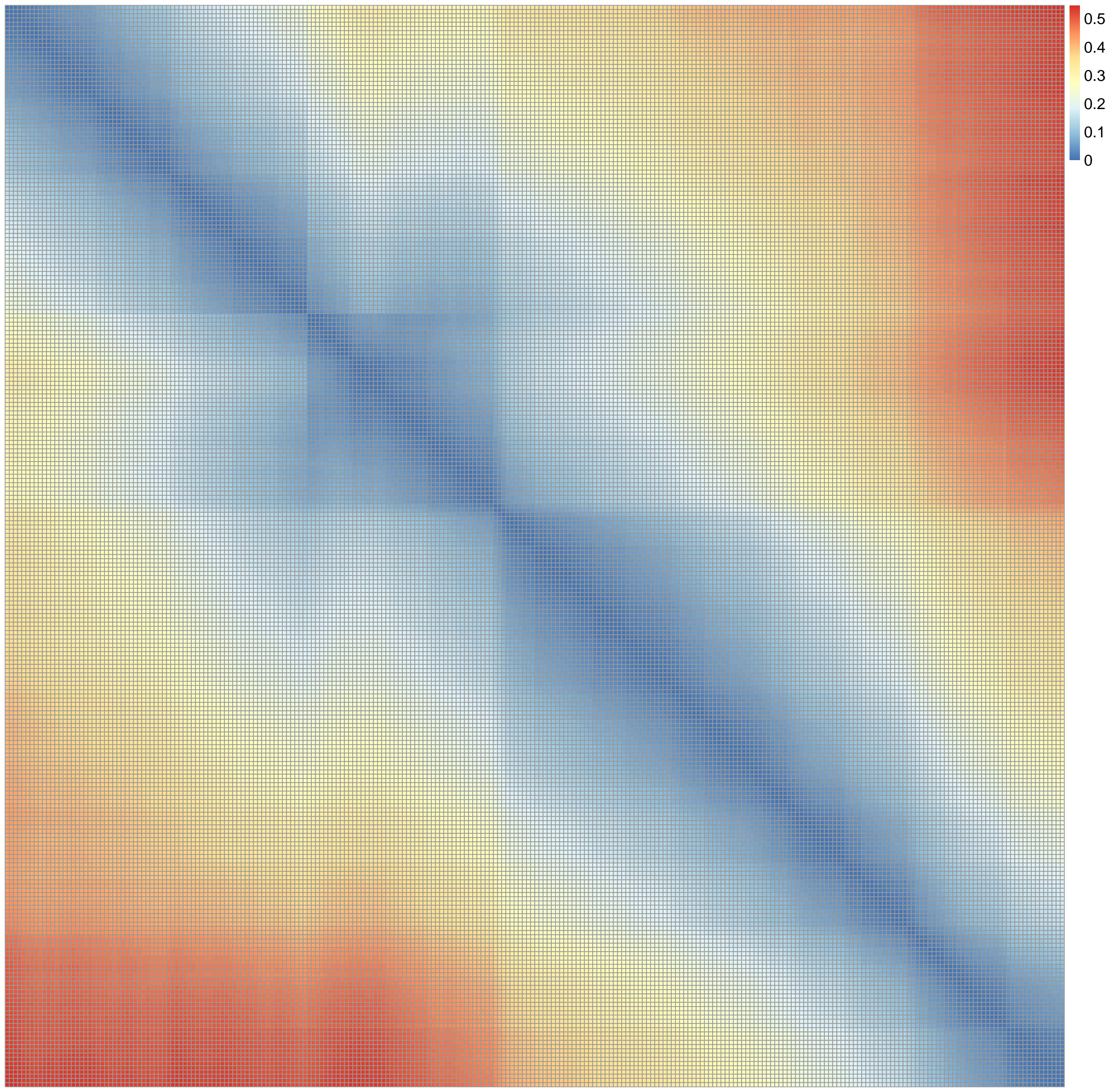

Supplement: Supplementary file 12 [file DataSheet11.pdf]

Lba11

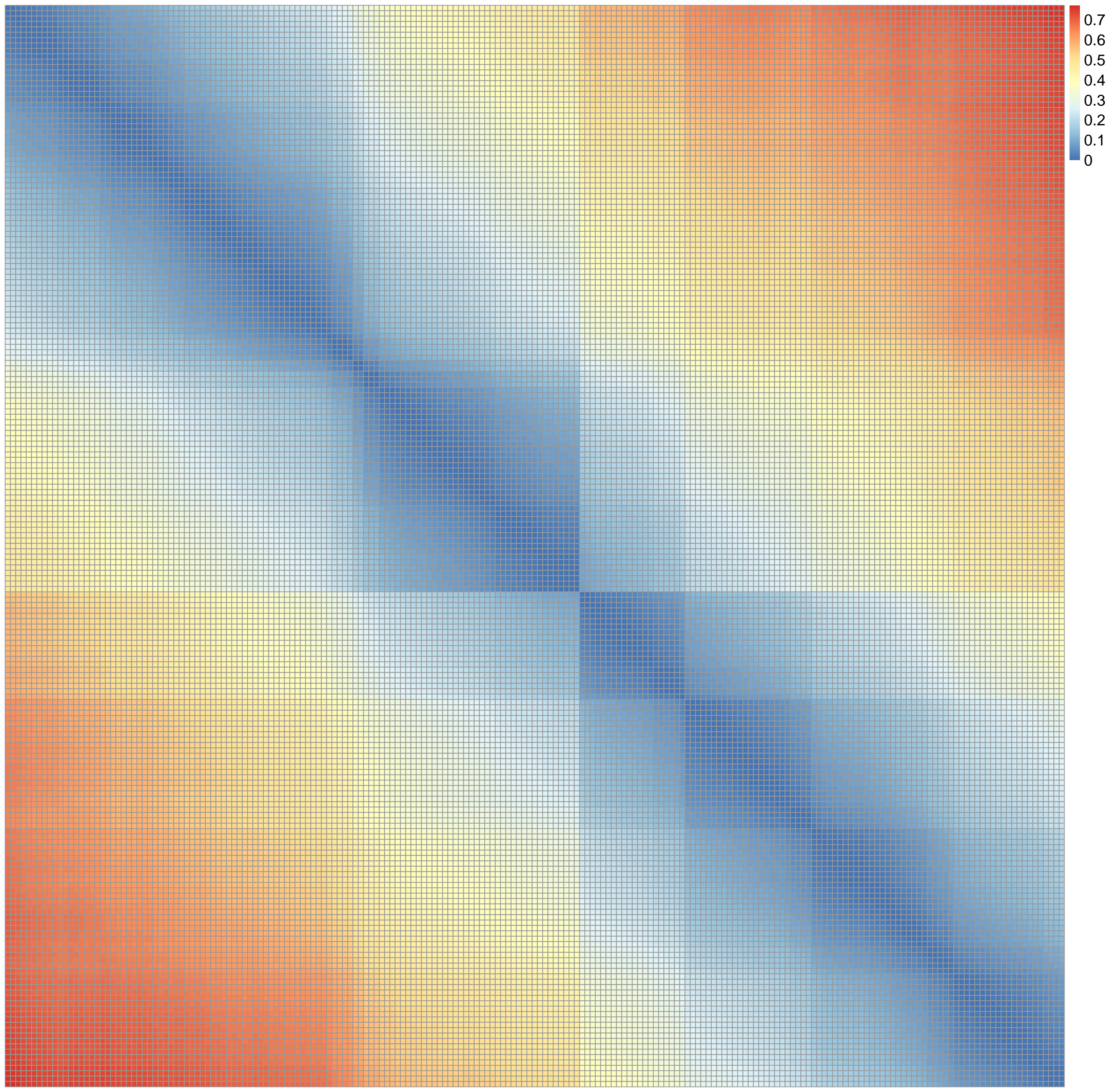

Supplement: Supplementary file 13 [file DataSheet12.pdf]

Lba12

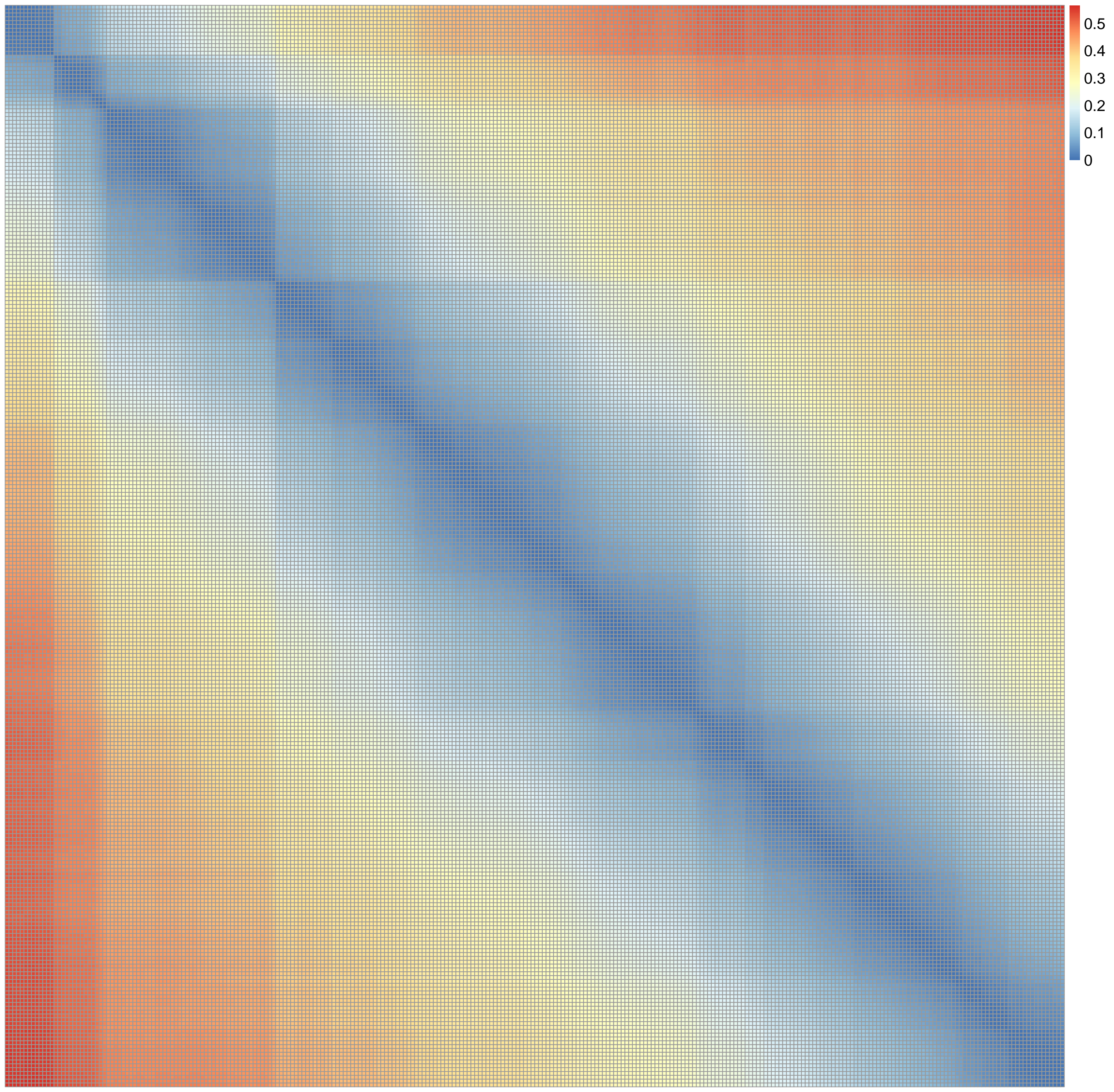

Supplement: Supplementary file 14 [file DataSheet13.pdf]
